# The perceived value of human-AI collaboration in early shape exploration: An exploratory assessment

**DOI:** 10.1371/journal.pone.0274496

**Published:** 2022-09-12

**Authors:** Andrés Arias-Rosales

**Affiliations:** Department of Mechanical Engineering, Carnegie Mellon University, Pittsburgh, PA, United States of America; University of Liverpool, UNITED KINGDOM

## Abstract

As a vital element of early shape exploration, divergence can be time-consuming and challenging, with iterative cycles where idea fixation and creative blocks must be overcome for fuzzy ideas to be fully expanded and understood. Despite interesting tools that have been developed for this purpose, some important challenges remain, as it appears that many designers still prefer simple freehand sketching and tend to defer the use of computational tools to later stages. This work presents an exploratory assessment of the perceived value of a new tool, Shapi, developed to assist early shape exploration by addressing some of the pitfalls reported in the literature. Shapi is envisioned as an autonomous assistant that provides local and global shape variations in the form of rough sketches based on an initial human sketch and interactive cycles. These shape variations are What-If scenarios and cognitive facilitators that may spark new ideas or enable a deeper understanding of the shape and the identification of interesting patterns. Shapi’s capabilities are explored in a diverse set of case studies with different purposes: nine implementations in industrial design, three in graphic design, and five with open-ended artistic purposes. These implementations are then used in a survey about initial perceived value in which the majority gave high ratings in terms of exploration (75.5% ≥ 4 out of 5), interpretation (83.7% ≥ 4), adaptation (77.6% ≥ 4), value (73.5% ≥ 4), creativity (69.4% ≥ 4), and general interest in the tool (79.6% ≥ 4). This work brings insight into promising functionalities, opportunities, and risks in the intersection between artificial intelligence, design, and art.

## 1. Introduction

**Fig 1:**
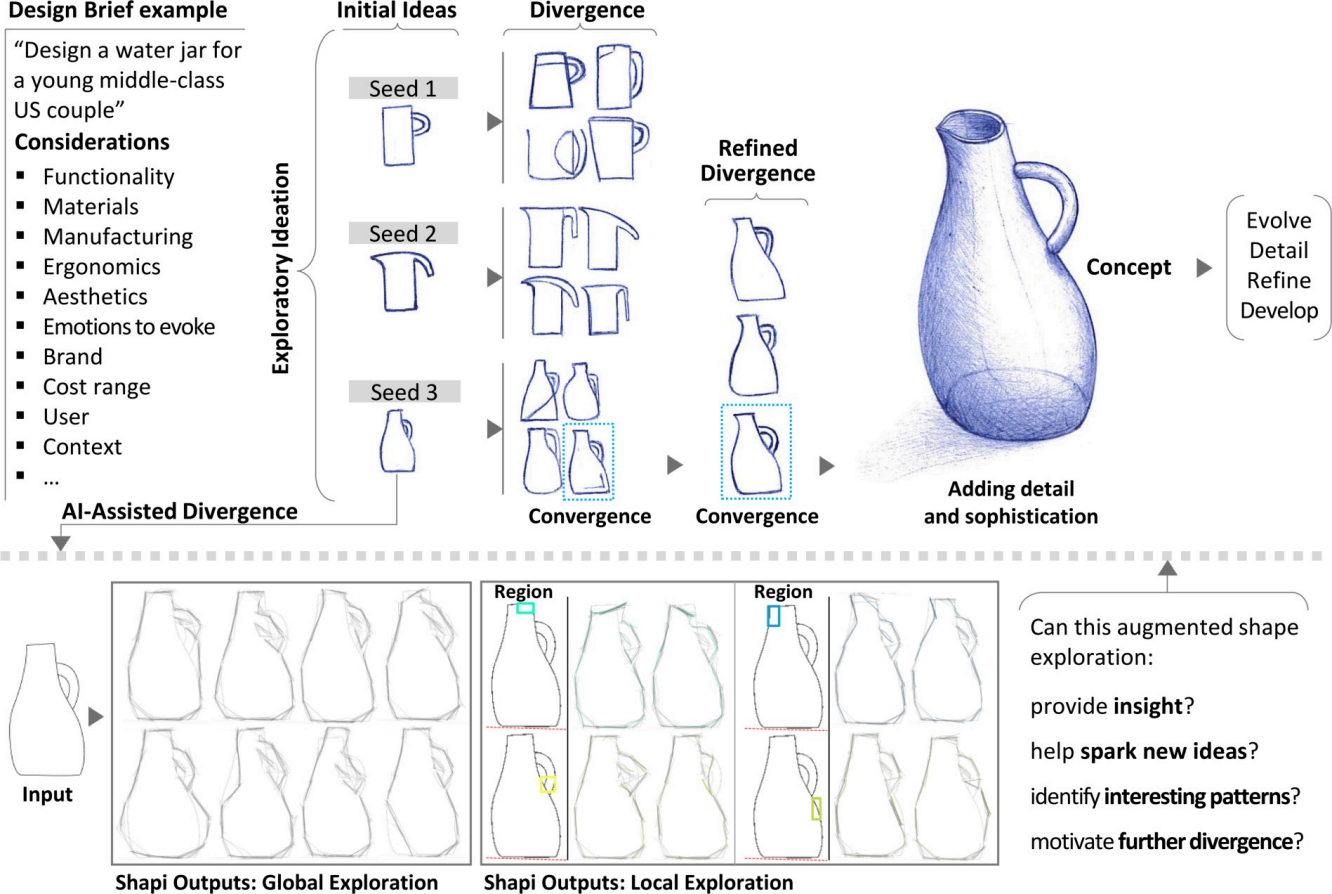
A simplified example of the divergence-convergence cycles of shape exploration for the conceptual design of a water jar. The basic shape can be progressively refined with rough sketches before developing the concept further. This work proposes an AI (Artificial Intelligence) tool, *Shapi*, for assisting shape exploration divergence as a process parallel to human sketching. Shapi generates variations around a given ‘Seed’, where Global variations can be used to catalyze insight or ideas about the overall shape or particular details anywhere in the contour, while Local variations can be used for more systematic inspections of the different shape regions.

In the early design stages, designers usually go through divergence-convergence cycles [1]. Sketching with rough lines [2] is a vital tool in this exploration for representing what is in the designer’s head, and for reasoning and discovering through the act of sketching itself; it is a reflective conversation [3] through ‘thinking sketches’ [4]. From the design brief [5], there is a general idea of what kind of product is being targeted and interconnected information about the user, context, and state-of-the-art. The designer integrates this information and may start by sketching a first set of embodied vague ideas. These ‘Seeds’ are explored by generating variations while trying not to lose their essence (Fig 1). By adding, deleting, deforming, or replacing elements [6], exploratory sketching enables cognitive processes of abstraction, emergence, and reinterpretation [7] that assist creativity in design [8].

During this diverging shape exploration, designers acquire a better understanding of the ‘emotional promises’ [9] of the Seeds and their variations. This understanding results from employing comparative reasoning among a broad range of alternatives and navigating the design space with What-If scenarios [10, 11]. Along with shape divergence is shape convergence, whereby patterns that catch the interest of the designer are identified, selected, and reinterpreted in successive or parallel cycles [12–15]. For convergence, the selection of shape alternatives is often based on multiple criteria, including some that are qualitative and subjective in nature [16]. Accordingly, shape styling may begin as a fuzzy exploration of appealing general shapes indirectly guided and constrained by high-level associations in the creator’s mind, while the details can be developed in successive stages [17, 18].

Exploratory divergence requires considerable effort and often involves repetitive procedures [19, 20]. Generating shape variations with enough breadth and depth can be time-consuming and challenging, especially for novice designers [20]. Although efficiency itself may not be a key issue in conceptual design, it can be an important limitation because creators often use heuristic strategies (e.g., dateline, exhaustion, number of sessions) to end early divergence and move on to more detailed and convergent phases (see Sec. 2.2). Also, creators can get stuck in a limited space of familiar solutions, which can result in a creative blocker [14] or design fixation [21, 22]. Thus, there has been a growing research interest in assisting conceptual divergence and in tools for making early shape exploration easier and more accessible [23, 24]. There is a variety of interesting computational tools that have been proposed for this purpose, from new Computer-Aided Design (CAD) implementations to Genetic Algorithms (GA), Shape Grammars, and deep learning models (Sec. 2.2). However, it appears that important challenges still remain regarding the adoption and acceptance of such tools, as more traditional techniques like simple hand sketches have been reported to be the dominant medium for early exploration in practice [25–27]. Some of the limitations that explain the slow adoption may be that most available tools like CAD softwares are well suited for detailed design stages [28], but can lead to premature convergence and hinder free exploration. For instance, such tools may use overly-defined shapes [25] that lack the characteristic flexibility, fluidity, and ambiguity intimately related to early creativity [29]. Another limitation is when a tool becomes negatively distracting in early exploration due to technical difficulties [30], drifting away too much from the ‘normal’ workflow, or involving too much low-level geometric manipulation [31]. Lastly, excessive automation, such as a tool that generates full shape solutions, may cause pushback from practitioners due to a sense of lack of control and of being replaced rather than assisted by the tool [32].

The growing research interest in developing and studying tools that assist early shape exploration (see Sec. 2.2) is often motivated by the critical role that divergence can play (e.g., providing What-If scenarios, facilitating comparative reasoning, and enabling a deeper understanding of the shape). Other motivations include the practical difficulties (e.g., repetitiveness, the cost in time and effort, skills that can be hard to develop, idea fixation, and creative blocks) and the potential consequences of insufficient divergence (e.g., quality and creativity issues in more advanced stages of shape definition). There are challenges that remain with the available tools, their adoption, and their perceived value. Based on relevant published literature (see Sec. 2), it seems promising to pursue and study tools for assisting early divergence with the following characteristics: that can generate a broad range of global and local variations, that receive a human initial embodied idea (Seed) and explore around its shape without losing the original essence, that involve open-ended human-AI (Artificial Intelligence) interaction, that allow for controlled randomness to introduce potentially surprising variations that spark new ideas and insight, that are easily adaptable (e.g., not requiring an extensive specialized training dataset), that produce variations that avoid premature convergence (i.e., with some degree of ambiguity, incompleteness, and roughness), and that are less disruptive of the familiar workflow of free sketching. Accordingly, the goals of this work are:

1. To develop a tool for human-AI collaboration in early shape exploration based on the abovementioned directives
2. To explore the capabilities of this tool in a range of implementations with different shapes and purposes
3. To perform an exploratory assessment of the initial perceived value of such a tool

To address these goals, the theoretical background and empirical evidence reported in other studies are used for building the foundations (Sec. 2) and methods (Sec. 3) of this work. A new tool, *Shapi*, is proposed (see Fig 1, Sec. 4 and S1–S3 Appendices) and its versatility and capabilities are explored in 17 case study implementations in industrial design, graphic design, and artistic exploration (Sec. 5). A description of the tool and some of the inputs and outputs from the case studies are used in a survey to assess the perceived value (Sec.6). The resulting scores are analyzed with descriptive and inferential statistics and the open feedback is examined with sentiment analysis (Sec. 6). Lastly, the main insights, limitations, and future work are discussed (Sec. 7) and summarized (Sec. 8). The original survey, the survey responses, the full output sheets from the case studies, and the files to run the algorithm are openly available at [33].

There are many interesting questions that can be raised about the general approach proposed in this work. For instance, is a tool like Shapi flexible and capable enough to be useful in real-world design cases? is the proposed workflow perceived as more ‘natural’ compared to alternative approaches? is this level of automation ‘acceptable’? can such tools overcome idea fixation and creative blocks? is the proposed output style appealing and ambiguous enough? are all this aspects dependent on age, education, or occupation? Moreover, the interactions between potential users and the tool itself introduce additional complexities and questions about the interface design, the hardware, and the spaces involved. Each of these questions and more may be analyzed extensively in future studies about Shapi or similar tools and they can become research projects on their own, so reaching definitive conclusions on all those fronts is beyond the scope of this project. Also, evaluating the user experience of Shapi is not among the goals of this work. The focus is on exploring the strengths, weaknesses, and limitations of the general approach based on internal implementations and assessing how potential users perceive the value of such a tool based on the concept of the tool itself and its capabilities illustrated by demonstrative examples.

Initial perceptions, namely, before using the tool directly, are of special interest here because they can bring insight into how potential users perceive: the problem of early shape exploration; the need for automated assistance or an AI collaborator; the appeal of Shapi’s general characteristics based on first impressions; the potential usefulness of the examples if the person imagines herself exploring the same shape; and the emotions, questions, and ideas that emerge after being exposed to this content. This work is an exploratory assessment that brings insight into these initial perceptions to better understand whether there is enough interest and potential in the general approach. If first impressions of the general concept are not interesting or if they evoke negative emotions, then potential users are less likely to be willing to use it on their own initiative and there may even be a strong push back. Initial perceptions are important as potential facilitators or barriers to adoption and it seems reasonable to assume that at least some potential users narrow down their options based on general interest before they start systematically testing and learning how to use tools. It is worth noting that initial perceptions may sometimes be misleading and they may change after using an actual application software, which is why questions about user experience and comparative performance involving the user are important for future work. Nevertheless, an exploratory assessment of initial perceptions can help identify what aspects, questions, and challenges are more relevant and this can motivate further technological developments, as well as future studies with more focused questions, larger scales, and involving more practical considerations.

Therefore, the main contribution of this work consists of the insights obtained about the general capabilities, focus, and type of outputs of a tool like Shapi contextualized in the proposed workflow, including encouraging, negative, or risky observations, as well as potential limitations and paths forward. The development of Shapi is a technical contribution to the computational creativity and interactive design communities. This tool provides capabilities with a promising focus that resulted in mostly positive ratings in the survey that was conducted. This work can bring new understanding into the intersection between AI, design, and art, which can be valuable for design researchers and educators.

In the interest of disambiguation regarding the use of ‘AI’ here, the ‘Artificial’ refers to the computer hardware-software employed, and the ‘Intelligence’ refers to tasks that would require intelligence if performed by humans [34], without implying that the tool is ‘truly intelligent’ from a human perspective. ‘Design’ is used here mostly to refer to the early conceptual phase in matters of industrial design. Regarding the use of ‘designer’ or ‘potential user’, the specific title is not as important as the assertion that this person is involved with the process of early shape exploration in some way and aesthetics and styling are at least some of the priorities motivating such exploration. Lastly, ‘case study’ is used here to refer to ‘a particular instance or case that may be analyzed or used as an example to illustrate a thesis or principle’ [35]. A case study in this work is when Shapi is implemented to explore a particular shape with a particular purpose and this is used as a demonstration and example of its capabilities, as well as an opportunity to examine the strengths, weaknesses, and limitations of the tool. A very similar use of this term can be found in closely related studies [14–16, 36–40] in which the capabilities of a tool are explored through internal implementations.

## 2. Background

This section discusses the theoretical foundations that inspire the characteristics of Shapi as a tool and workflow, the case studies, and the survey.

### 2.1 Thinking sketches: A reflective conversation

As a crucial aspect of the theoretical framework of this work, this section discusses the sketching process as a reflective conversation [3] for exploration and discovery. Sketching is key to the creation of concepts [14], not only for externalizing and recording ideas [41], but also as a means to explore them [42], as thinking tools [41] and cognitive facilitators [43].

Sketching creates opportunities for reinterpretation and emergence that favor creativity in design and art [14, 44]. Sketching can be described as a conversation between the creator and the visual representations being materialized [45]. As sketches start taking place, the curves are perceived by the author and this can spark new ideas and elicit reinterpretations, the discovery of emerging patterns and associations, and provide new insight into the often vague shapes that were originally in the author’s mind. This new insight can be used to continue an unfinished sketch, to transform (‘move’) it, or to generate new shape variations. This occurs in cycles of ‘see-move-see’ [42] and ‘reflection-in-action’ [30]. Oxman [44] proposed ‘the thinking eye’, a conceptual framework that intimately relates visual cognition and visual perception with emergence during conceptualization. Similarly, Creative Segment Theory models the process of ideation through sketching, where a Creative Segment is a loop of idea generation, expression, and visual feedback that involves brain, hand, and eyes [41].

Exploratory sketches, also known as idea-sketches, are usually freehand rough and ‘fresh’ representations that contrast with presentation-sketches, which usually have unambiguous and detailed contours and are used in later stages [45, 46]. Ambiguity is key in exploratory sketches because it makes it easier for the triggering of different ideas and for the author to concentrate on the general character and aesthetics of the shape, while temporarily suspending judgment on the details and some of the engineering aspects [45]. When a sketch with ambiguous contours is subject to perception and reflection, different specific shape solutions can emerge from one same sketch. Also, if a shape region or detail is not critical in the early conceptual phase, it can remain ambiguous while the author focuses on the rest. Ambiguity has even been described as a prerequisite for emergence and the novelty that empowers creativity [44]. Nonetheless, the degree of ambiguity is a trade-off; if the shape is too vague, it may not be evocative enough, or relevant aspects of the concept may get lost.

During exploratory sketching, it is common for the creator to focus on a few key leading curves [47, 48] that capture a major portion of the shape’s character and the author’s intent, such as profiles, sections, and reflection lines [17, 31, 49]. For generating shape variations, creators can implement general transformation rules to the leading curves, such as outline transformation, structure transformation, substitution, addition, deletion, cutting, and view change [14]. Shape variations can be valuable because they allow the creator to assess a given contour or set of contours on a more intuitive relative basis, e.g., ‘this one looks better’, ‘this curve evokes more dynamism’, or ‘this shape seems more practical’. Since these kinds of assessments are highly subjective, it would presumably be more difficult to evaluate them on an absolute basis. Moreover, sometimes a creator may like or dislike a particular shape but not quite know why until it is compared to slight variations in a tinkering process. Other times, a creator may not know whether a basic shape (Seed) has good potential until it is explored further through transformations.

**Fig 2:**
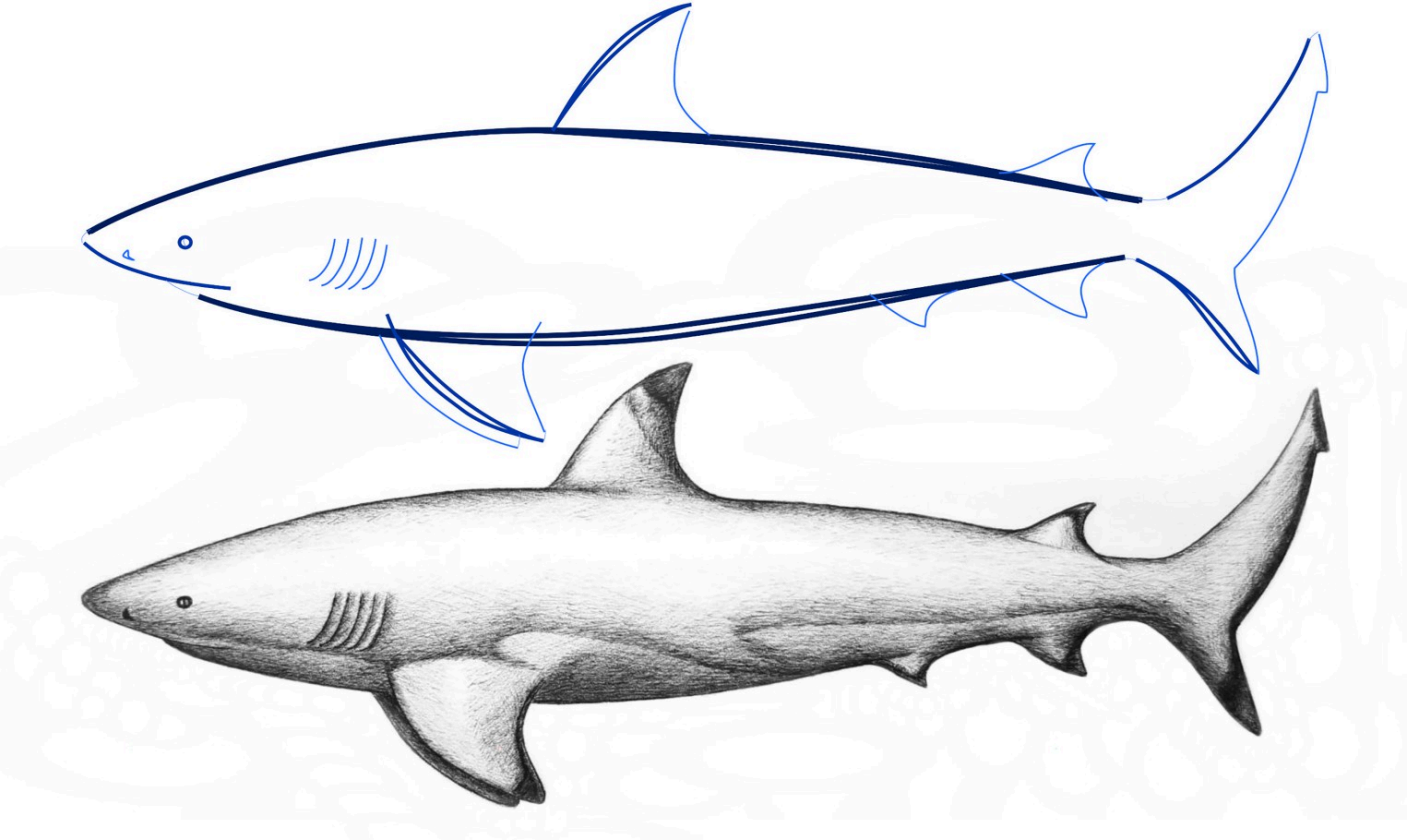
Drawing a blacktip shark, sketching as a reflective conversation. The curves (top) are thicker and darker the more important they are for establishing and discovering the general character of the drawing.

In a firsthand example of sketching as an act of both creation and discovery, Fig 2 describes the drawing of a blacktip shark. This technique involves using a ballpoint pen to start exploring very tenuous and somewhat ambiguous general curves that get progressively cleaner and more well-defined as details of small features, volume, and texture are added. At the beginning, it is difficult to form a complete and crisp mental image of the shark, so its shape is progressively ‘discovered’. The first main contour lines (displayed as thicker curves in Fig 2 - top) are tenuously drawn with slight variations until the general proportions seem ‘right’. It is easier to judge something already materialized than to create it from scratch, so every curve facilitates the next one and new details are progressively added as a response in this reflective conversation.

### 2.2 Related works: Computer-aided shape exploration

Previous work has investigated computational tools and methods for supporting shape exploration in an early conceptual phase. This phase usually deals with solution spaces that are underdefined and where alternatives are judged based on a combination of both objective and subjective criteria. If all the possible solutions were to be systematically explored, this would often lead to a ‘combinatorial explosion’, which motivates designers to approach the problem through heuristic strategies [30] that lead to good-enough solutions in limited amounts of time and effort [34]. This results in the ‘time versus completeness dilemma’ [50]. To address this, designers use intuition, high-level associations based on references and previous experience, random stimulation, parallel and sequential cycles of divergence-convergence, and stopping criteria such as fixed time, number of sketches, or mental exhaustion. Such characteristics of the conceptual phase make it challenging to develop tools that can effectively support this process without negatively limiting the natural workflow [18, 32]. It has been noted how computational tools can easily break and restrict the creative flow in conceptual design due to distractions and technical difficulties related to the use of digital interfaces, as well as a common lack of flexibility in the generation or transformation of concepts and a lack of ambiguity in their appearance [45, 46]. The development of computational tools for supporting sketching in the early design stages is a challenge that has elicited considerable interest in the design research community [42].

Although tools involving CAD models have traditionally been used for more advanced and detailed stages of the design process, they have also been proposed for conceptual design. Alcaide-Marzal, Diego-Mas, and Acosta-Zazueta [14] proposed a method where the features of a product are divided into bounding volumes, which are then populated from a set of 3D shapes that can be modified interactively. Khan and Awan [20] used a Jaya algorithm with a weighted-grid-search to generate CAD model variations within a viable space that can be established explicitly, autonomously, or interactively. Pernot et al. [18] introduced free-form deformation functionalities to facilitate a more direct and intuitive control of global and local variations in CAD surfaces. Hsiao and Wang [36] proposed an environment with semantic control based on mapping words, 3D models, and geometric transformations. Also supporting semantic-driven tools, Giannini, Monti, and Podehl [31] discussed how low-level geometric manipulation can be limiting and distracting for designers. Prats et al. [42] argued that CAD tools have struggled in conceptual stages because of their use of precise geometries as opposed to more fluid representations. As a potential solution, Rusák and Horváth [11] developed an approach where a vague interval model of a 3D shape can be used to instantiate specific shape solutions with global or local variations.

Randomness has been integrated into different tools designed to support ideation because it can lead to potentially surprising solutions that go beyond preconceived ideas. Nonetheless, Belmonte et al. [51] suggested allowing for an interactive balance between generative randomness and control. For instance, Krish [52] implemented constrained random variations of CAD models for shape exploration. Genetic algorithms and Shape Grammars have been used to integrate randomness within the ideation process [51].

Dang et al. [53] explored product shapes with Shape Grammars interactively conditioned by a Probability Density Function. Pugliese and Cagan [54] examined the feasibility of using Shape Grammars to capture brand identity in concept variations. Seeking a more intuitive manipulation of 2D curves during styling, Cheutet et al. [17] mapped properties of curves to Shape Grammar operators. Shape Grammars have also been used as a generative method within a GA that optimizes design solutions [37].

Genetic Algorithms have been instrumental in addressing the ‘time versus completeness dilemma’ in conceptual design. Roy, Jared, and Mussa [16] integrated a GA with CAD software to support free-form exploration by optimizing spline smoothness. Shieh, Li, and Yang [40] fitted regression models related to Kansei Engineering to predict affective responses as part of a GA fitness function. One key challenge is determining a fitness function that is computable yet rich enough. Stanley and Lehman [55] described how algorithms with clear objectives may fail to generate interesting images, so they suggest exploring open-ended objectives without predetermined solutions. Accordingly, GAs with interactive fitness functions that include human subjective input have been proposed to explore architectural models [15], phone designs [38], wine glass shapes based on revolved splines [39], lamp models for stimulating the ‘mind’s eye’ [13], conceptual designs of a car console [56], and car silhouettes based on Bézier splines [12]. In addition to an interactive GA, one study [57] employed a neural network to approximate the designer’s implicit aesthetic while generating cartoon faces. Moreover, for exploring variations based on human input, the GA-WA (Genetic Algorithm-Weibull Arias) algorithm [58] presented functions for biasing the initial population of a GA toward an intuitive initial genotype.

Different methods have been used for representing sketches in these algorithms, such as silhouettes based on Bézier splines [12], Fourier decompositions [49, 59], Shape Grammars [37], or digital curves fitted with a GA from scanned drawings [60]. ‘ShadowDraw’ [61] provides guidance in the form of shadows based on matching sketches. ‘Sketch-RNN’ [62], trained on recordings of stroke-based human drawings, suggests shape completions or alternate versions. Lopez, Miller, and Tucker [63] used deep learning algorithms to generate rough sketches and compared them to human sketches with promising results. Lastly, using the ‘QuickDraw’ dataset in the Forma Fluens project, Martino et al. [64] analyzed superimpositions of thousands of people drawing the same common objects and found convergent representations that capture the essence of these entities and the ambiguity from different interpretations.

Based on previous research, some of the aspects that seem to be desirable for a shape exploration tool are: intuitive, flexible, with controlled randomness, highly interactive, open-ended, and capable of generating a broad range of global-local variations. It is also suggested to avoid excessive automation that may restrict human creativity and, instead, focus on hybrid approaches that support deep human-AI collaborations [32].

## 3. Methods

### 3.1 Development of the tool

The target functionalities are established based on the directives summarized in Sec. 1, which are inspired and motivated by relevant published literature (Sec. 2). These include a rough description of what the tool should be capable to do and the type and style of inputs and outputs. Then, the proposed workflow is defined (Sec. 4.1), namely, how the tool would ideally be used in a generic design scenario in which a shape is initially explored through a human-AI collaboration. The proposed workflow includes some considerations about the ideal user experience.

The target functionalities and the idealized human-AI workflow are then used as the guidelines for developing the algorithms of the tool, which are described in detail in Sec. 4.2 and the S1–S3 Appendices. The methods and Python 2.7–3.7 libraries used in these algorithms include: the ‘*scikit-image*’ and ‘*matplotlib*’ libraries for processing and generating raster images; the ‘*random*’ library for initializing stochastic procedures; the ‘*numpy*’ library for algebraic operations; the ‘*sklearn*.*linear_model*’ library for fitting linear regression models while characterizing boundaries and axes; the principles of a 2D Perceptron are used for enforcing symmetries and boundaries; K-Means Clustering [65] for finding and delimiting geometrical segments in the shape; a Finite-Difference scheme [66] for identifying ‘high-interest’ points; cubic Bézier curves as the basic components for shape representation; a Genetic Algorithm and its functions [67] for partially fitting Bézier curves; and a ‘Nudge’ function proposed here to increase and control shape variation.

The functional modules of the main algorithms depend on a series of heuristic procedures and the parameters that control them (see S1–S3 Appendices). Hundreds of combinations of heuristics and parameters are tested seeking to find a set of practical functionalities that approximate the target capabilities. When the parameters of metaheuristics such as Genetic Algorithms [68] are being defined, identifying optimal values can be considerably complex and time-consuming, and tends to be very specific to a particular set of cases, so there is a considerable risk of over-fitting. Instead, the goal here is to find ranges of parameters that work reasonably well for a diverse range of cases. Different heuristics and parameters are iteratively tested with a central implementation case, i.e., the jar depicted in Fig 1, and then those settings are further explored with other case studies involving different shapes and purposes (Sec. 3.2).

### 3.2 Internal case studies: Purpose, shapes, and areas of application

As discussed in Sec. 3.1, it is important to explore the capabilities of the tool with a diverse range of shapes and purposes to examine how potentially generalizable the algorithmic heuristics and parameter ranges are and whether the resulting practical functionalities approximate the directives that motivate this work (Sec. 1). Case studies are used here as internal implementations in which the general approach is used to explore a particular shape with a particular purpose. Each case is an opportunity to study the technical feasibility of the approach and identify strengths, weaknesses, and practical limitations. Moreover, these case studies provide a set of example demonstrations of inputs and outputs that are then used as part of the survey to evaluate perceived value (Sec. 3.3). The use of internal implementations with the purpose to verify and explore the capabilities of an approach that is related to shape exploration appears to be a relatively common practice among some of the studies that motivated this work [12–16, 18, 20, 36–40, 49, 52–54, 69].

A set of case studies are conducted in Industrial Design (Sec. 5.1), namely, implementations in which the general shapes of consumer products are explored. The chosen products are a jar, a hand-axe, a mug, a perfume, a dispenser, a water bottle, a liquor bottle, a lamp shade, and a car. Similar studies based their implementations on seven products [14], four products [20], three products [18], or two products [15, 52, 69], and most focused on one product [11–13, 17, 31, 32, 36–40, 49, 53, 54, 56]. Nine products are explored in this work for an additional range of opportunities to verify Shapi’s capabilities. The product selection considers a range of different complexities in terms of how shape details relate to potential interpretations and function. For example, the hand-axe and lamp shade are expected to be more ‘forgiving’, as a broad range of details would still be plausibly related to the main function (e.g., some kind of surface for gripping and a sharp end for chopping, or a wide surface for light diffusion). Conversely, a car silhouette is expected to be more complex in the shape-to-function relationship. The types of products chosen are all inspired by external shape explorations (cited in Sec. 5.1) and some of the products are similar to the ones explored in other studies: a jar-like vase [40], perfume bottles [14], wine glasses [18, 20, 39], a Coca-Cola bottle [37], lamps [13, 14, 20], and automotive [12, 17, 31, 36, 49].

The main focus is on Industrial Design applications. Nonetheless, since the Shapi algorithms can receive abstract shapes and symbols as inputs, three additional case studies are conducted in Graphic Design to explore whether this is a promising area of application. The cases consist of a badge, a logo, and a texture pattern (Sec. 5.2). Furthermore, another five case studies are conducted to explore whether the Shapi approach has potential for purposes that are more open-ended, divergent, and artistic (Sec. 5.3), including the emergence of shapes that resemble wings, cartoon faces, flying animals, and aerial vehicles. The emergent patterns may be comparable to other studies that have explored cartoon faces [57] and airplane shapes [53]. In total, this work includes 17 internal case study implementations.

### 3.3 Survey: General design

As discussed in Sec. 1, the purpose of the survey is to assess how potential external users perceive the value of a tool like Shapi based on the concept of the tool itself and its capabilities as illustrated by a series of demonstrative examples from the case studies (see Sec. 3.2). Several closely related studies also generated a series of shape variations internally (i.e., without the participation of external users) and then conducted various kinds of evaluations based on external perceptions about such variations [20, 40, 49, 54]. For instance, it appears to be a relatively common practice to assess whether images produced with generative algorithms are perceived as realistic or plausible based on survey respondents who did not participate in their creation [70, 71].

The survey consists of the following sections:

1. A brief description of Shapi’s purpose, capabilities, and proposed workflow (based on Sec. 4.1). With this section, the participants are exposed to the concept of Shapi as a tool and as a potential collaborator.
2. The participants are asked ‘How interesting would you find a tool like Shapi?’ based on the brief description and is requested to provide a score from 0 to 5 to rate this initial **interest**. This score is expected to depend on what participants imagine the tool might be like and their preconceptions about the general problem of early shape exploration, the use of computational tools, and automation in general.
3. The participants are asked to provide anonymous information about occupation, undergraduate and postgraduate studies, and age group. The goal is not to seek a complete characterization of perceptions segmented along these factors and with evenly distributed subgroups. Instead, as this is an exploratory assessment, the goal of gathering this information is to analyze whether the survey scores present interesting and seemingly consistent patterns of perception that depend on occupation, studies, or age. Such preliminary patterns can motivate questions and future studies that look into them more systematically and at a larger scale.
4. The survey displays a set of examples taken from the Global and Local Explorations in the case studies (six industrial design cases, three graphic design cases, and four artistic cases). In all examples, the respondents are shown the input Seeds and Shapi-generated variations, but no human reinterpretations. This is because the goal is for participants to focus on Shapi’s potential capabilities and imagine themselves continuing the human-AI conversation based on Shapi’s variations. They are explicitly requested to imagine this while they observe the demonstrations. Also, during the artistic demonstrations, the participants are repeatedly asked (rhetorically) ‘What do you see?’ to motivate them to search for interesting open-ended patterns. During a pilot test in which the case studies were presented to two professors and a group of graduate students, it was observed that attendees tended to focus too much on the human reinterpretations if shown and they were easily misunderstood as made by Shapi even if it was stated that they were human-made. Thus, the illustrative demonstrations were simplified in this regard. This pilot test was also used to determine the number of demonstrations to display in the survey.
5. The participants are requested to provide 0–5 scores to rate how they feel about Shapi in terms of the following constructs (as defined here and in the survey):
  - **Exploration**. Construct (based on the concept of exploratory creativity [72]): It generates variations within a delimited solution space. This is evaluated because it relates to the core target functionality (see Sec. 4).
  - **Interpretation**. Construct: The variations preserve the essence (type of product and general form) of the initial sketch (for Industrial Design cases). This construct is important because even if the tool is good at generating variations (i.e., high in Exploration), if the essence of the input is lost, then something else is being explored instead of the seed, which is the intended focus for Industrial Design applications.
  - **Surprise**. Construct (based on [72]): Are there non-obvious variations? This construct is an important part of an effective divergence process because, if variations are too obvious, then they are presumably less likely to be perceived as evocative or valuable idea catalyzers and cognitive facilitators (see Sec. 2.1).
  - **Ambiguity**. Construct (based on [44]): Can the variations have multiple specific solutions / interpretations? As discussed in Sec. 2.1 and 2.2, some degree of ambiguity is suggested in the literature as a potential way to overcome idea fixation and premature convergence.
  - **Adaptation**. Construct: The tool works for different uses and types of products and shapes. This construct is related to the target functionalities because a tool is presumably less likely to be perceived as potentially useful or valuable if it is perceived as not adaptable enough.
  - **Style**. Construct: How much do you like the style of the sketches proposed by the tool? This construct is evaluated because the aesthetic appeal of variations may be related to perceived value and interest.
  - **Value**. Construct: The tool allows exploring the potential of a shape and proposes interesting and potentially useful variations. This more general construct is directly related to the motivations for developing a tool like Shapi (see Sec. 1). In other words, this is the kind of value that Shapi should ideally provide to potential users. This construct is expected to be related to the constructs in (a), (b), (e), and (f), so the participants have already reflected on some of the elements of this broader construct before evaluating the value of Shapi as a general concept. The intention is for the participants to gradually build a more global perception. Also, some amount of redundancy can be used to analyze consistency and look for unexpected relationships and trade-offs.
  - **Creativity**. Construct (based on [73]): The ability to explore and diverge in novel and useful ways while preserving the original essence of what is being explored. As with (g), this is also a more general construct that is expected to be related to the others to some extent.
6. The participant is asked again ‘How interesting would you find a tool like Shapi?’. The question about **Interest** is asked right after the initial description and again after a series of demonstrations and reflections elicited by the process of evaluating the metrics (a-h). Thus, comparing the Interest before and after is an opportunity to analyze whether such demonstrations meet the initial expectations. Also, the trends in Interest (after) can be compared to trends in other perceptions to examine whether the overall interest is related to the specified constructs.
7. Lastly, there is a section for open feedback where the respondents can make any comments, suggestions, and freely discuss their perceptions after being exposed to the proposed workflow, a series of demonstrations, and evaluations.

Regarding the number of participants used to evaluate some aspect of a generative approach, similar studies have involved various kinds of surveys or workshops with a sample size of: 150 [54], 95 [71], 62 [51], 60 [40], 41 [41], 30 [56], 26 [31], 12 [20], 10 [45], 8 [49], 2 [57], and 1 [11, 32]. There seems to be a trade-off between how many participants can realistically be obtained and how involved the survey is. For instance, two of the analyzed studies with the most participants involved only one binary question, namely, whether a generated shape was a Harley-Davidson or not [54], and whether emulations of retinal vasculatures seemed realistic or not [71]. Another challenging trade-off to navigate is whether to include only a very specific group of participants (e.g., particular groups of students [41, 45, 51, 56]) or a more diverse distribution. This work’s survey is not as involved as an in-depth and hands-on workshop. Yet, it is comprised of a series of sections involving various activities, from reading to reflecting on incomplete human-AI ’conversations’ and rating perceptions and interest. In pilot tests, it was estimated that responding to this survey takes approximately 5–10 minutes. Additionally, since this an exploratory assessment of perceptions, the goal is to include participants from a range of design and art-related backgrounds and occupations, from students to teachers and practitioners. Based on similar studies, the expected difficulty of the survey, the exploratory nature of this work, and the practical limitations of the project, 50 participants is defined as the target sample size (49 responses were obtained; Sec. 6).

### 3.4 Survey: Methods of analysis

In total, every participant is requested to provide 10 numerical scores. Each score is limited to integers from 0 to 5. Since these are distinct categories that are mutually exclusive and ordered, the scale can be classified as at least an ordinal scale [74], which may often be described by metrics like mode and median. Additionally, if the numerical values related to these scores and the distances between them are assumed to be meaningful and consistent, then this scale could be treated as an interval scale, which may be analyzed with additional metrics like means and Standard Deviations (SD). This distinction is not necessarily clear-cut, it involves important trade-offs, and has been debated for decades with multiple schools of thought [74–76]. Velleman and Wilkinson [75] warn about potentially ‘oversimplified’ views that could lead to losing important information when data that could be reasonably described in terms of an interval scale is forced to be ‘demoted’ to ordinal scale. In his influential book [76], Van Belle states that long-held conventions about ordinal versus interval scales can sometimes be ‘unnecessarily restrictive’, and that the distribution of values can be more important than the strict definition of the scale of measurement.

In this work, there is no intention to dive further into this debate or take a hard stand. Instead, following the suggestions of some of the sources that have discussed the various perspectives [74–76], a more pragmatic and exploratory approach is chosen. For instance, the difference between a score of 4 and 3 seems numerically meaningful and it does not seem completely unreasonable to compare it to the difference between a score of 2 and 3. Hence, to describe the central tendency of the scores, both means and medians are used. Similarly, both SD and Variation Ratio (VR) [77] are used to describe the dispersion of the scores, and both Person’s r and Spearman’s Rank-Order [76] are used to describe correlations. The pragmatic approach here is to look at both groups of metrics and examine whether they are consistent and agree on the same general trends and relationships.

Regarding the analysis of the open feedback, the ‘*wordcloud’* library [78] in Python 3.7 is used to graphically analyze the hierarchy of concepts that are more frequent and salient. Moreover, sentiment analysis is used to estimate the Polarity of every feedback sentence; a continuous variable ranging from -1 (most negative sentiment) to 1 (most positive). Polarity is obtained by averaging the results from the ‘*Pattern’* tool in the ‘*TextBlob’* library [79] and the ‘*Vader*’ tool [80] in the Natural Language Toolkit (*NLTK)* suite of libraries. These are both widely popular sentiment analysis tools [81].

For inferential tests of hypotheses, Monte Carlo schemes are used as practical alternatives to parametric methods because they do not assume underlying distributions of residuals and are more flexible with the type of scale [82]. For a 5% significance level, it has been suggested to perform at least 1,000 Monte Carlo simulations [83]. To better approximate exact permutation tests of hypothesis [84], this work uses 100,000 Monte Carlo simulations for every test of hypothesis. The following inferential questions are tested:

- **Regarding scores** (Interest before and after and metrics a-h in Sec. 3.3): The null hypothesis is that those scores are drawn from a discrete uniform distribution (*H*_0_: *scores*~*U*(0,5)) and that the means and medians describe the central tendency of a uniform random variable. The Monte Carlo scheme simulates a population of scores in groups of 49 (i.e., the sample size as the data) by sampling from a discrete uniform distribution. The one-tailed *P*-value for the mean of a given score is the number of times that a simulated mean is equal to or greater than the observed mean, over the total number of simulated groups [85]; and this is repeated in the case of medians.
- **Regarding Polarity:** The Monte Carlo scheme here is almost the same as with scores. However, since Polarity is a continuous variable, two null hypothesis are tested, namely, that Polarity values are drawn from a continuous uniform distribution (*H*_0_: *Polarity*~*U*(−1,1)) or from a normal distribution with mean 0 and the same SD as the one observed in the data (*H*_0_: *Polarity*~*N*(0,*SD*)).
- **Regarding Interest Before vs. After:** The null hypothesis is that both distributions of Interest scores come from the same distribution and, thus, have the same mean and median (e.g., *H*_0_: *μ*_*Before*_ = *μ*_*After*_). The Monte Carlo scheme repeatedly and randomly reassigns the scores between the Before and After groups and calculates the differences between means and medians. The two-tailed *P*-value is the number of times that the absolute value of a simulated delta between means or medians is equal to or greater than the one observed, over the total number of data reassignments [82].
- **Regarding Correlations:** The null hypothesis is that there are no linear relationships (*H*_0_: *r* = 0). For every two groups being correlated, the Monte Carlo scheme repeatedly and randomly changes the order of the values within each group and calculates the simulated correlation metrics. The two-tailed *P*-value is the number of times that the absolute value of a simulated correlation metric is equal to or greater than the one observed, over the total number of data reshufflings.

### 3.5 Survey: Ethics statement

The survey was shared online with design and art students, teachers, researchers, and practitioners, explicitly stating that it was an evaluation for academic purposes. They were encouraged to voluntarily fill out the survey and share it at their own discretion, in their own time, and without any type of monetary incentive, coercion, psychological pressure, or manipulation. The participants voluntarily and freely consented to access the survey, fill it out, and submit it. Anonymity was protected throughout the process, and the only data that was stored was information that was explicitly provided and only after participants had completed and submitted their responses. This study does not involve minors, any kind of medical or physical procedure, or any significant psychological strain (e.g., 5–10 mins looking at shapes and answering simple and short questions based on their perceptions). No monetary gains have been obtained at the expense of the participants or their data, and the original survey and anonymous results are made openly available at [33]. This work was conducted without any direct funding or institutional resources beyond the author’s own time and years-long dedication. For all the reasons above, no ethics committee was deemed necessary.

## 4. Shapi: An AI tool for assisting divergence in shape exploration sketching

This section describes the development of Shapi, a new tool designed to fulfill the directives described in Sec. 1. The workflow proposed for Shapi (Sec. 4.1) is intended to support an intimate human-AI collaboration. The human creator is in the ‘driver’s seat’, providing the Seeds, interactively setting up the tool, identifying interesting patterns, selecting and reinterpreting shape variations, and integrating shape divergence into the general design context, where many more variables are considered. Shapi is an assistant that serves as an extension in breadth and depth (not a replacement) of the creator’s capability to explore a shape and facilitate divergence. How the tool works is described in Sec. 4.2 and S1–S3 Appendices.

### 4.1 Workflow: A human-AI collaboration

**Fig 3:**
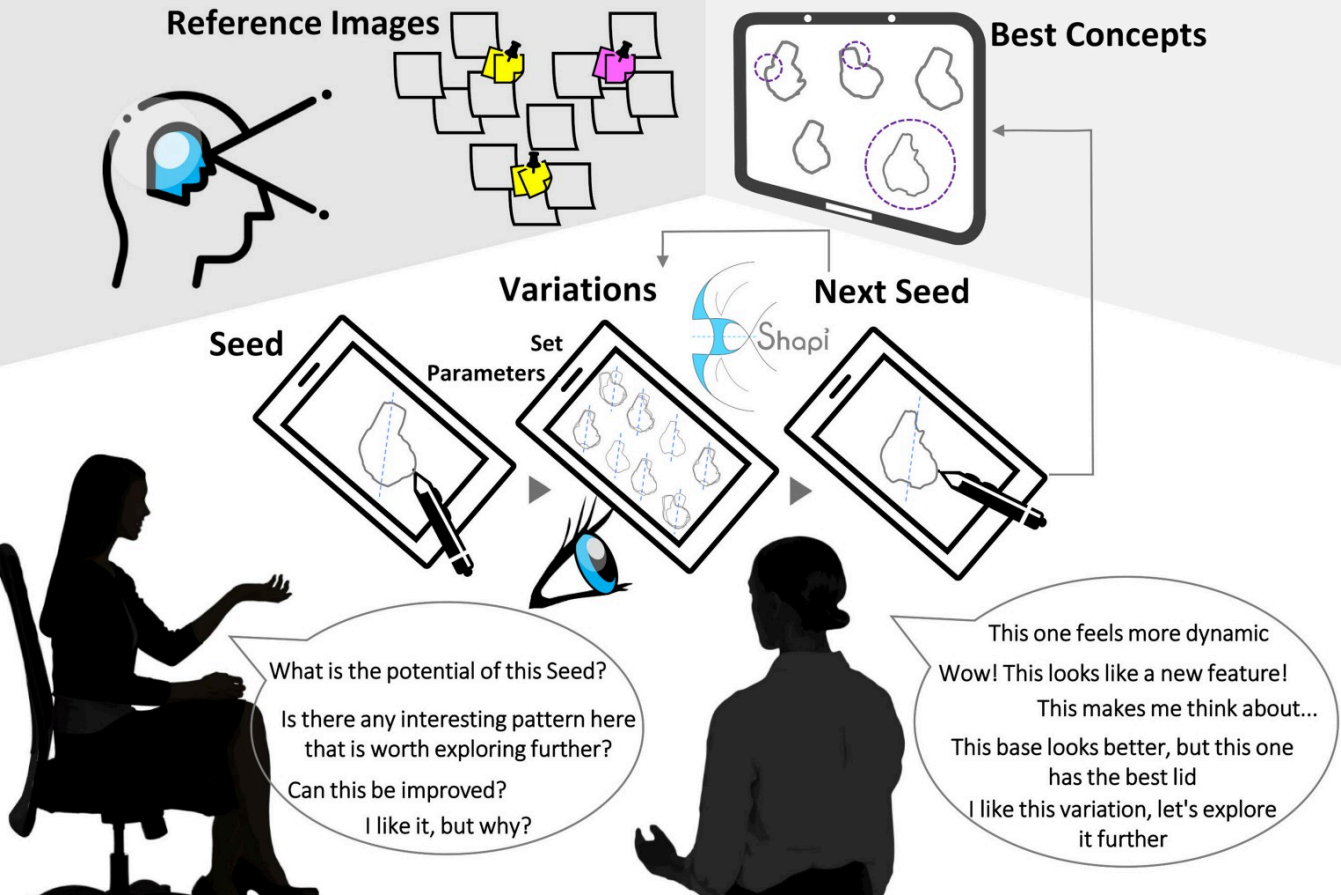
Suggested workflow: Using Shapi in a human-AI collaboration for early shape exploration.

Shapi is a tool designed to support a human-AI collaboration for shape exploration in the early conceptual phase. As shown in an example scenario in Fig 3, the collaboration begins with the user drawing a Seed shape. After setting the parameters of the algorithm, Shapi is used to explore the potential of that Seed by generating local and global variations (Fig 1). The user perceives these variations and reflects on the evolving shape (see the example reflections and questions in Fig 3). This reflective conversation may inspire the user to generate a new Seed after acquiring new insight, forming new associations, and discovering interesting emerging patterns. Reference images, mood boards, and the stored representations of the best concepts so far can be used to guide the conversation. The new Seeds that become the input of the next cycle may be: a direct selection from the previous exploration, an evolved version of one of the previous variations, an integration of several variations, an isolated feature, or perhaps an entirely new idea.

This process of divergence-convergence can happen in successive or parallel cycles that ideally lead to the progressive refinement and understanding of the shape. At the beginning of every cycle, the user can interactively set the parameters of the algorithm to adjust the exploration breadth and how aggressive or divergent it is desired to be. Another way of interacting is that users can draw colored lines to indicate boundaries or symmetry axes. These interactions can determine whether the tool is used for shape refinement or for discovering new and potentially surprising patterns. The workflow should not be restrictive (e.g., forcing the user to expand on Seeds only through Shapi), and it should ideally be compatible with a hybrid approach where additional cycles with more familiar techniques can interact with digitally-augmented cycles.

The role of Shapi is to generate shape variations through the manipulation of elements that may be interpreted as lines of character, contours, or details. The user has the critical role of providing the Seeds, looking for meaning, and generating sketches as a response. **Shapi’s outputs are idea catalyzers, not final concepts**, so the final presentation sketches are meant to be human-generated.

This work aims to assess whether a tool like Shapi could be perceived as valuable for design academics, educators, and professionals. This section described a suggested real-life workflow. However, it is important to clarify that even though the Shapi algorithm has been developed and tested, there is not yet an application software ready to be used by the public. That will be part of future developments and evaluations but it is not part of the scope of this work.

### 4.2 How Shapi works

This section presents how the Shapi algorithm works to make the functionalities and interactions described in Sec. 4.1 possible. One key technical challenge is representing sketch Seeds in vector form. An input sketch must be translated into some kind of visual representation (phenotype) that is rich, yet simple enough to be controlled by some kind of encoded set of parameters (genotype). The phenotype should approximate the style of a rough evocative sketch with some degree of ambiguity, as opposed to a completely defined shape with clean and definitive contours. This is because the purpose of these phenotypes is to inspire ideas and possible reinterpretations, not to deliver final concepts. The next challenge is diverging in potentially meaningful, surprising, and valuable ways. There should also be a form of interactive control for regulating how close the variations stay to the Seeds versus how divergent they are, from ‘refined’ to ‘aggressive’.

**Fig 4:**
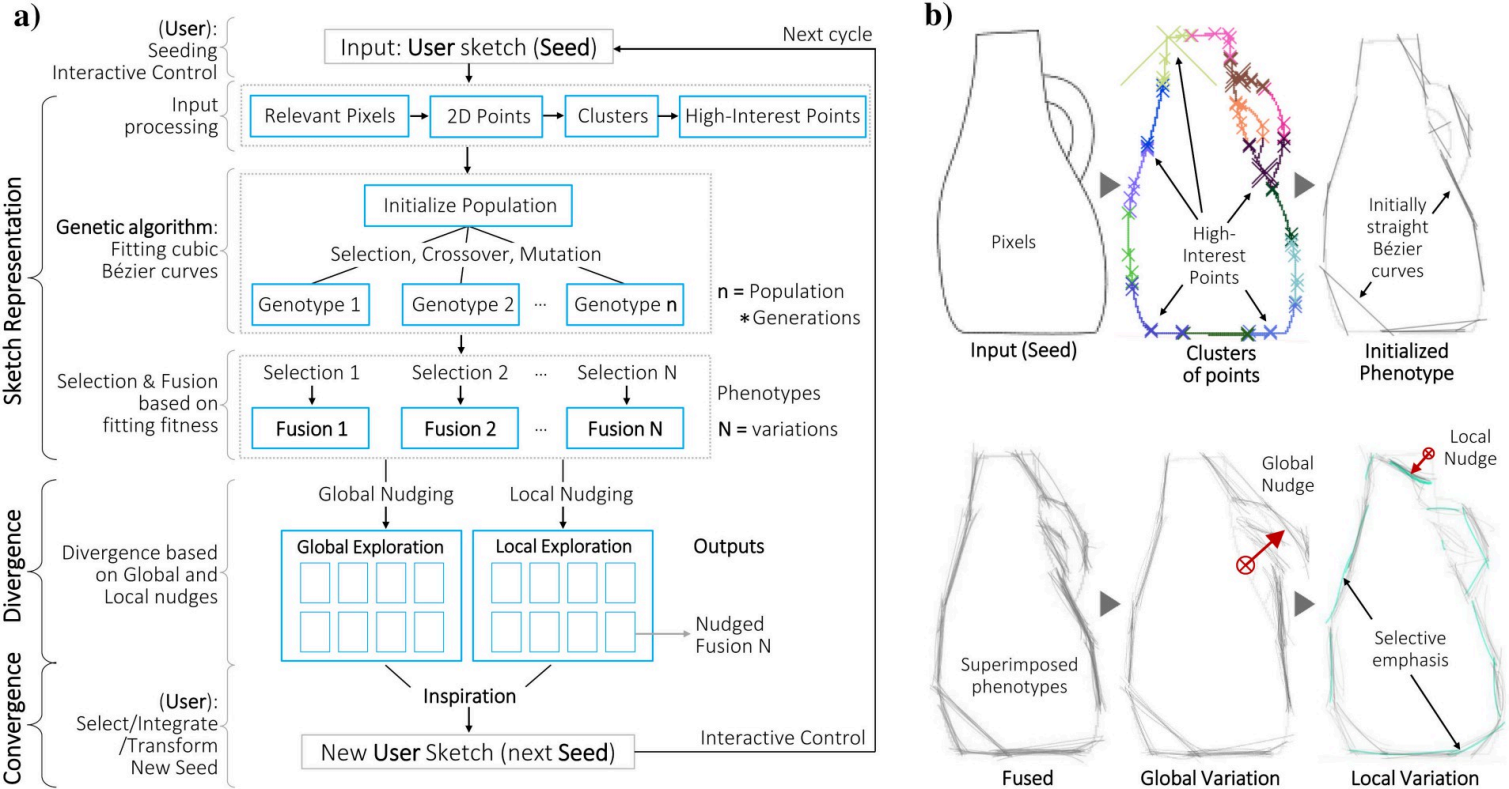
The approach. a) The general approach proposed to assist early shape divergence and exploration in a human-AI collaboration. Note that the human collaborator provides the Seed and the shape reinterpretation at the end of every cycle. b) An example shape starting with a given Seed and leading to suggested global and local variations with a rough sketch style.

The general approach is summarized in Fig 4(a) and exemplified in Fig 4(b). A sketch representation is obtained from a human input (Seed) by partially fitting sets of cubic Bézier curves with a Genetic Algorithm. A ‘Nudge’ function is used to implement stochastic local and global transformations that lead to shape variations. The outputs are organized and presented to the user (as in [33]), who interactively adjusts the settings for the next cycle of exploration. Sketch representation is explained in detail in S1 Appendix, divergence in S2 Appendix, and outputs and interactive control in S3 Appendix.

**Fig 5:**
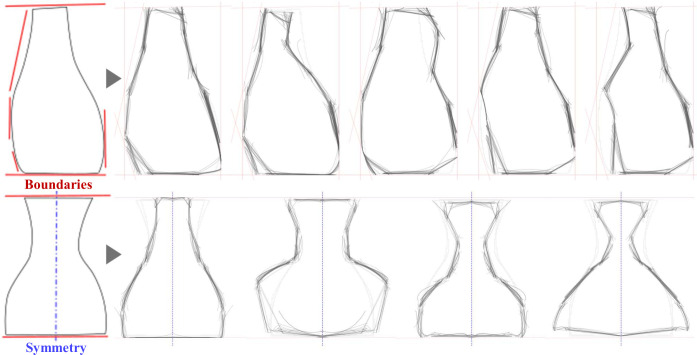
Examples of drawn boundaries and a symmetry axis to guide the exploration interactively.

As part of the interactive human-AI collaboration, the creator may draw boundary lines in red or a symmetry axis in blue. Fig 5 displays a few examples of the kind of global variations that can be obtained with Shapi using these functionalities. As these are global variations, deviations tend to be larger in magnitude, cover a larger area, and may happen at any automatically identified region of the contour.

**Fig 6:**
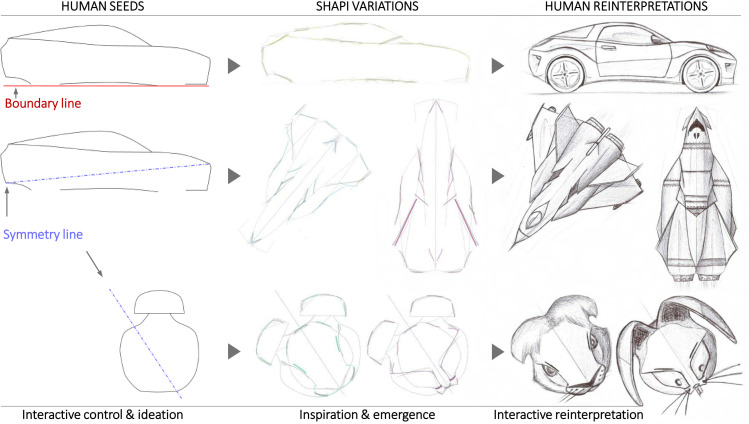
Explorations with different purposes. Input Seeds (left) are explored with Shapi (middle), and depending on the interactive controls, this may inspire reinterpretations as product concepts (top) or entirely new entities with divergent emerging patterns (bottom).

Some of the Shapi variations appear to display emerging patterns that may inspire the creator (Fig 6-center column), who can then generate a sketched reinterpretation in response (Fig 6-right). This response may or may not be more detailed and sophisticated depending on the creator’s purpose. For artistic purposes, any Seed can be explored with more aggressive settings and unusual symmetry lines, which may lead to open-ended reinterpretations that are not necessarily constrained by the original nature of the Seed (Fig 6-center and bottom rows).

## 5. Internal case studies: Exploring the tool

To explore the versatility and capabilities of Shapi, a series of demonstration case studies are conducted: nine implementations in industrial design (Sec. 5.1), three in graphic design (Sec. 5.2), and five with open-ended artistic purposes (Sec. 5.3). Only a few variations are displayed for every case, but the complete output sheets generated with Shapi can be found at [33].

### 5.1 Industrial design

Industrial design encompasses the products that are subject to visual judgment and appreciation in a meaningful way [31]. The following cases show Shapi implementations to explore the general shape of industrial products. For every case, an input sketch (Seed) is digitally traced based on third-party explorations of the same products. The purpose is to assess whether Shapi might be valuable in deepening and broadening the early shape exploration in these cases while preserving the essence of the Seed. These are not meant to be complete conceptual explorations, but rather exploratory inspections of the potential capabilities and limitations of the Shapi algorithm for a diverse range of products.

**Fig 7:**
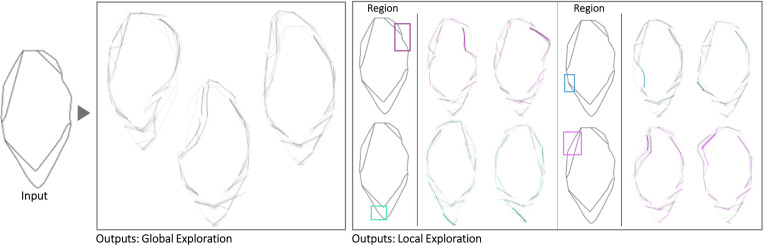
Some of the Global and Local shape variations generated with Shapi for the conceptual exploration of a hand-axe. The Seed is based on an exploration by a product & communication design student [86].

**Fig 8:**
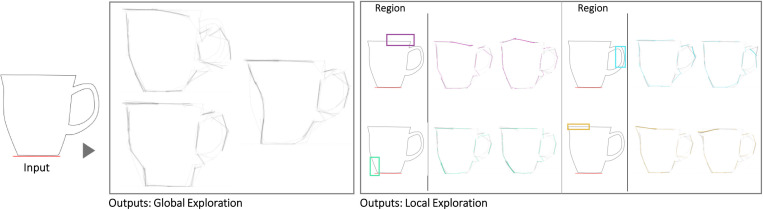
Some of the Global and Local shape variations generated with Shapi for the conceptual exploration of a coffee mug. The Seed is based on the silhouette of a design by Dunoon [87].

**Fig 9:**
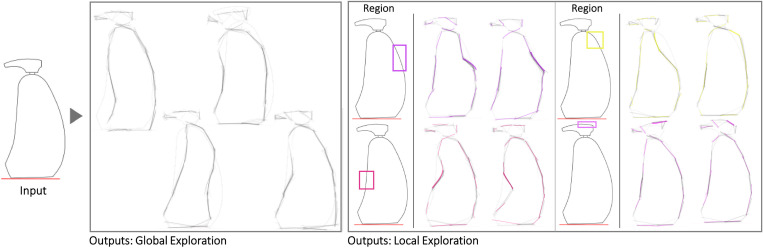
Some of the Global and Local shape variations generated with Shapi for the conceptual exploration of a soap dispenser. The Seed is based on an exploration by product designer & illustrator Von der Heyde [88].

The first case is a small hand-axe (Fig 7). The Shapi variations seem to preserve the original essence while also offering potentially meaningful shape alternatives. The automatic contour clusters (S1 Appendix) that guide the Local explorations seem to correspond to intuitive regions of interest, e.g., the tip, the handle region where the palm may be supported, and the region where the index finger can be placed for fine control. These Global and Local variations may inspire questions and ideas about ergonomics, usability, and mass balance. Figs 8 and 9 display the exploration of a coffee mug and a dispenser. The Global variations may inspire new shape directions with different aesthetics, while the Local variations can support a more systematic inspection of the different parts, such as the handle and the base.

**Fig 10:**
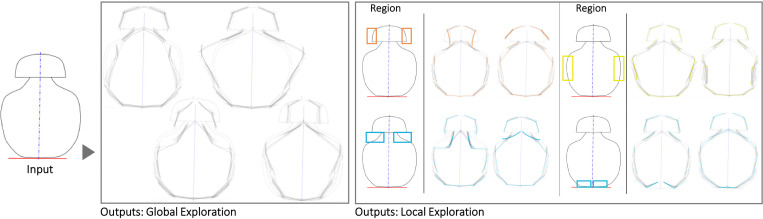
Some of the Global and Local shape variations generated with Shapi for the conceptual exploration of a perfume bottle. The Seed is based on an exploration by an industrial designer [89].

**Fig 11:**
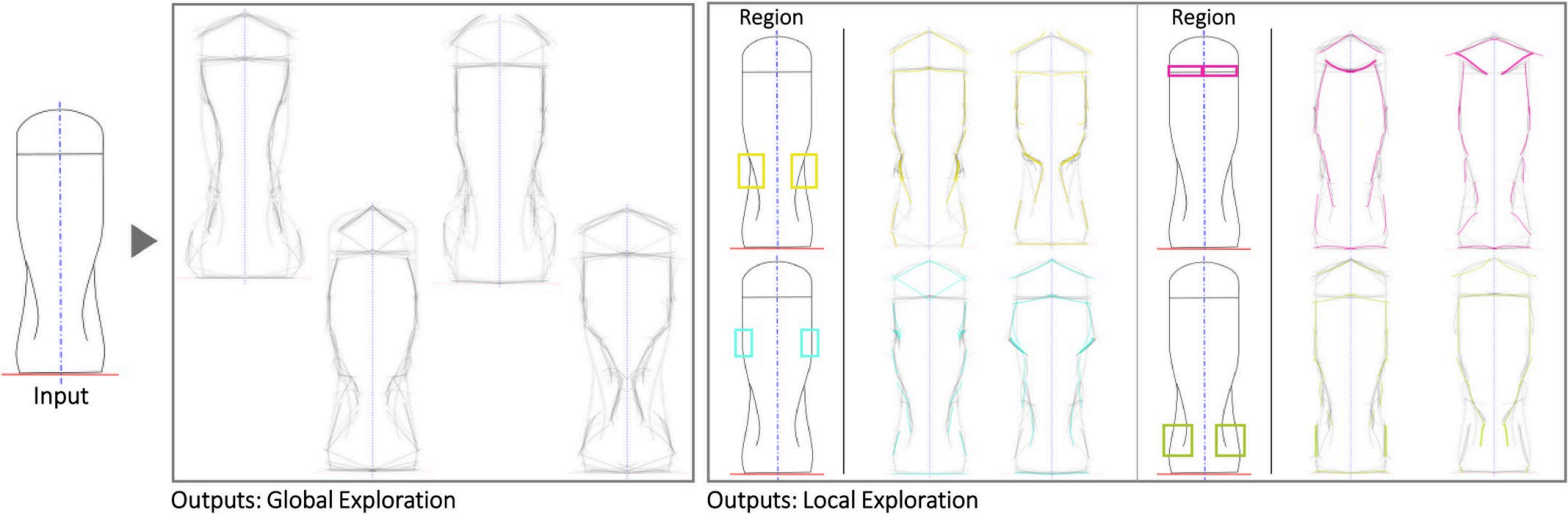
Some of the Global and Local shape variations generated with Shapi for the conceptual exploration of a water bottle. The Seed is based on an exploration by a product designer [90].

**Fig 12:**
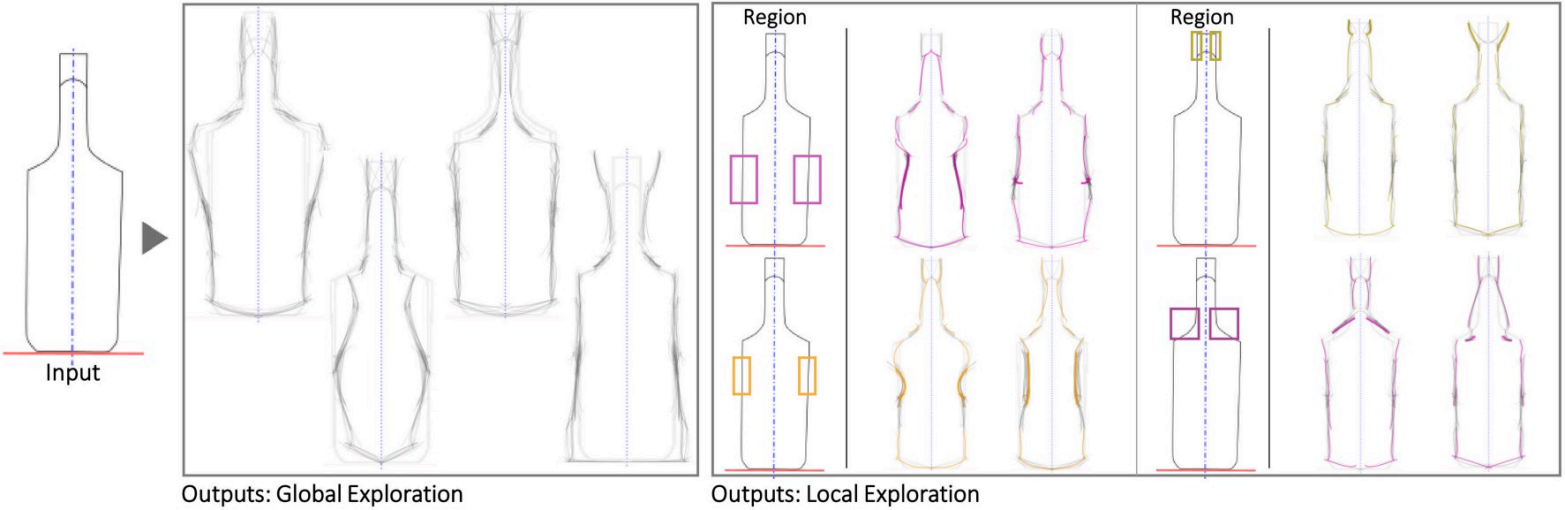
Some of the Global and Local shape variations generated with Shapi for the conceptual exploration of a liquor bottle. The Seed is based on an exploration by an industrial designer & entrepreneur [91].

**Fig 13:**
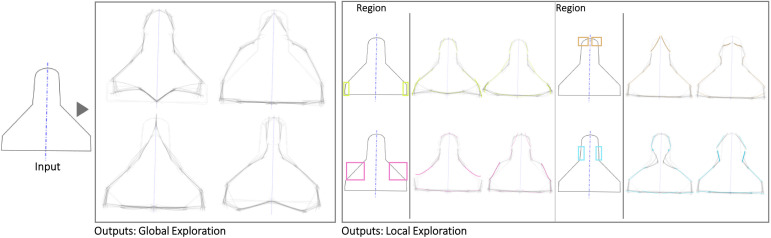
Some of the Global and Local shape variations generated with Shapi for the conceptual exploration of a lamp shade. The Seed is based on an exploration by an industrial designer [92].

Figs 10–13 illustrate four cases use the symmetry functionality. The water bottle case seems to be particularly illustrative of detailed texture variations in the region where the bottle would be grabbed and the interface between the lid and the main body. The liquor bottle case seems noteworthy because although the Seed is fairly generic, the variations seem to evoke a variety of bottle ‘personalities’ that could be valuable for different liqueurs or target users.

**Fig 14:**
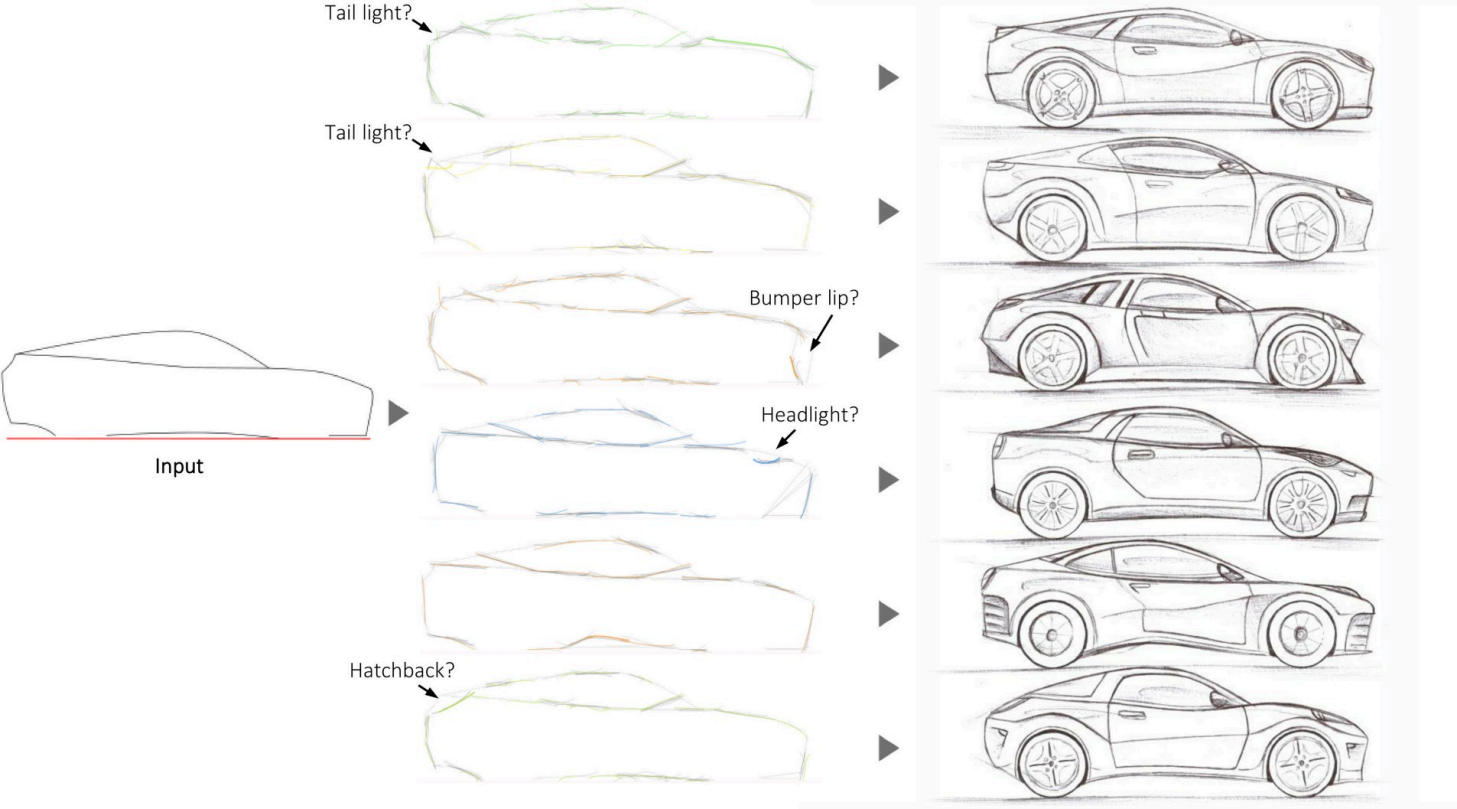
Shapi variations of the silhouette of a car (center column) where some characteristics may be considered ‘emergent’ features. On the right, some reinterpretations of how a designer might continue the ideation conversation. The Seed is based on an exploration by an interior photographer [93].

Fig 14 shows examples of the sketching ‘conversations’ that may result from a creator providing the Seed of a car silhouette, Shapi suggesting variations as a response, and the creator then reinterpreting and refining them as concepts. Due to the complexity of the shape, this is expected to be the most challenging case. Nonetheless, the shape variations seem valuable for inspiring localized ideas. It is worth highlighting that some of these local variations seemed to be interpretable as emerging features, such as lights and aerodynamic additions. Although the higher contour complexity hinders the quality and style of Shapi’s sketches, they may still catalyze interesting reinterpretations as part of the human-AI conversation.

Some of the shape variations in these cases suggest local features that would be unfeasible in practice, such as bottlenecks that would be too thin, or unstable bases. It is important to underscore that Shapi variations are not full design concepts. The designer is the one who integrates multiple criteria to generate feasible reinterpretations that may be inspired directly or indirectly by Shapi’s outputs. Also, as shown in Fig 14, the designer may use the rough and lose Shapi sketches as the backbone of more refined and detailed human sketches.

### 5.2 Graphic design

In the graphic design cases, three different abstract shapes are used as Seeds and explored with Shapi. Here, the objective is not to preserve the essence but to generate entirely new shapes and potentially surprising emergent patterns for graphic design purposes, such as inspiration for logos, emblems, sigils, or abstract decorative patterns to be used in backgrounds, textile printing, or textures. It is worth noting that graphic design is much more than just finding appealing abstract shapes; it includes the integration of high-level information, such as brand values or the particular emotions to evoke.

**Fig 15:**
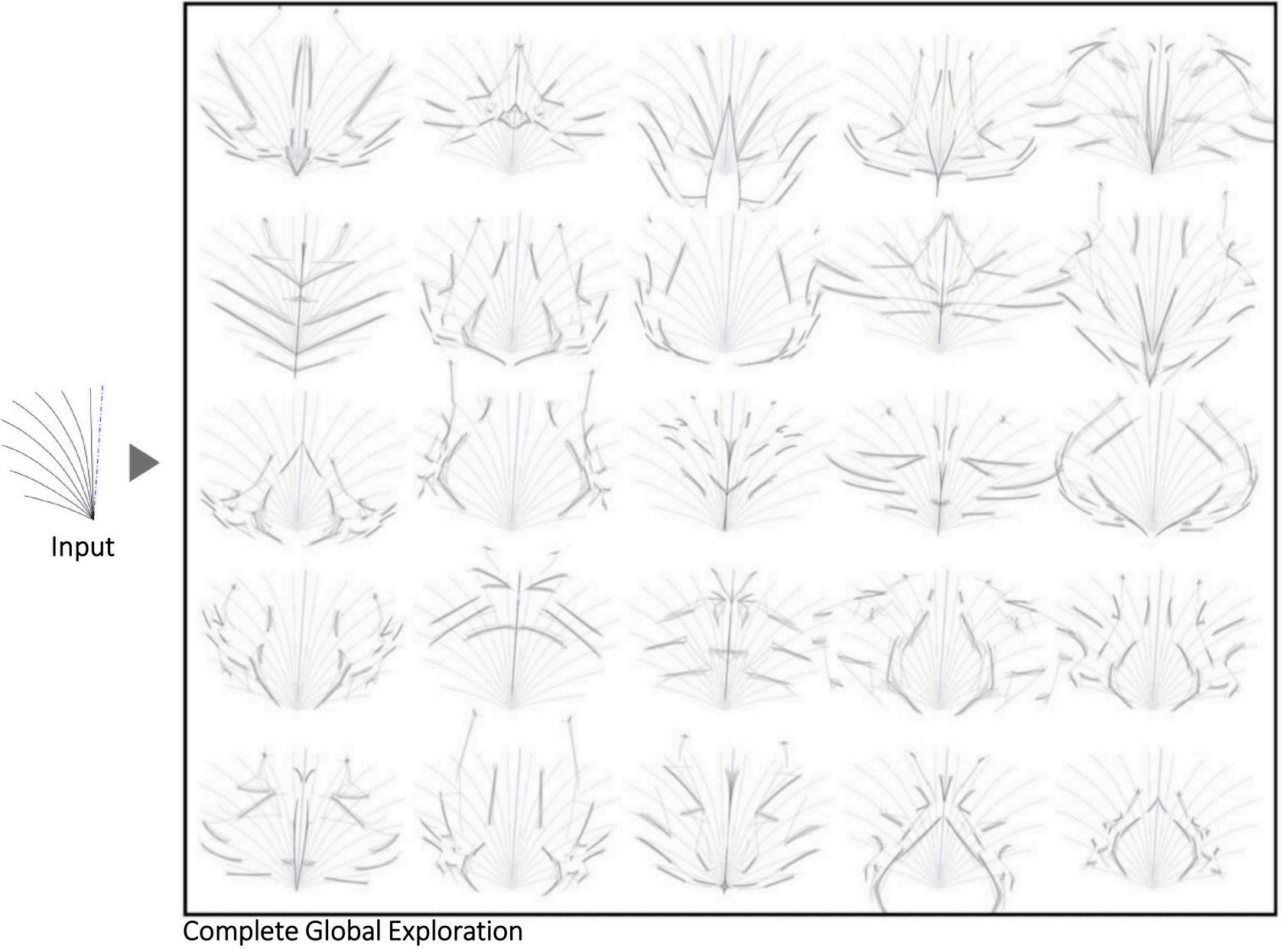
Example of an output sheet for the Shapi Global Exploration of an abstract input Seed.

**Fig 16:**
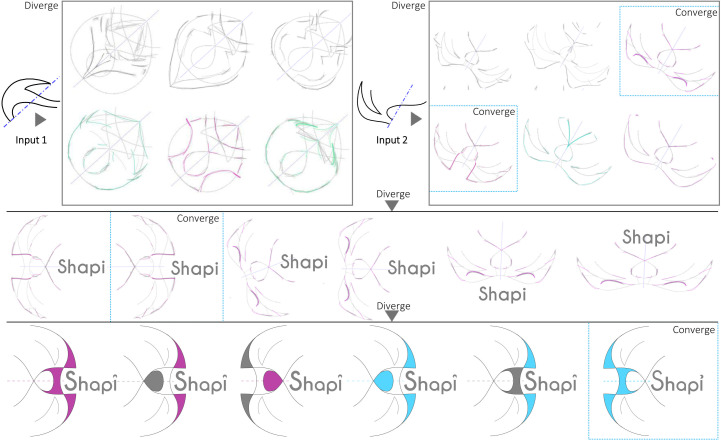
Two abstract Seeds are explored with Shapi. Several cycles of divergence-convergence result in the Shapi logo.

Symmetry axes are used in unconventional positions and Nudges are implemented with an ‘aggressive’ setting to increase divergence. Fig 15 demonstrates the capacity of Shapi to generate considerable shape divergence when that is the purpose. Such new shapes can be valuable for the creation of textures, decorative background patterns, or for inspiring more elaborate graphic entities. On the other hand, Fig 16 presents a more comprehensive exploration that results in the design of the Shapi logo. Two abstract Seeds are explored in parallel using Shapi. Then, two alternatives emerging from Seed 2 are selected (first convergence). In the next divergent phase, those two shapes are used as abstract pictorial marks in several logo combinations with a wordmark. One alternative is selected (second convergence) and refined further (final convergence). The shape variations emerging from Seed 1 seem valuable for the design of emblem-type logos. This is a simplified example that does not necessarily represent the full complexity of professional logo design.

### 5.3 Artistic exploration

The question of whether a creation ought to be considered art or not is an interesting discussion that is not in the scope of this work. Rather, the term ‘artistic exploration’ refers here to the purpose of the exploration more than the creation itself. In the previous cases (Secs. 5.1–5.2), shape variations are compared and interpreted with the guidance of relatively clear goals, such as establishing the silhouette of a car or finding the appropriate pictorial pattern for a particular logo. Conversely, the purpose here is to search for surprising emergent shapes that could be reinterpreted as entirely new entities. The intention is to assess whether Shapi can be valuable for discovering interesting new shapes that diverge greatly from the input. This is an open-ended exploration that is more prone to follow inspiration and emergence regardless of where it leads.

**Fig 17:**
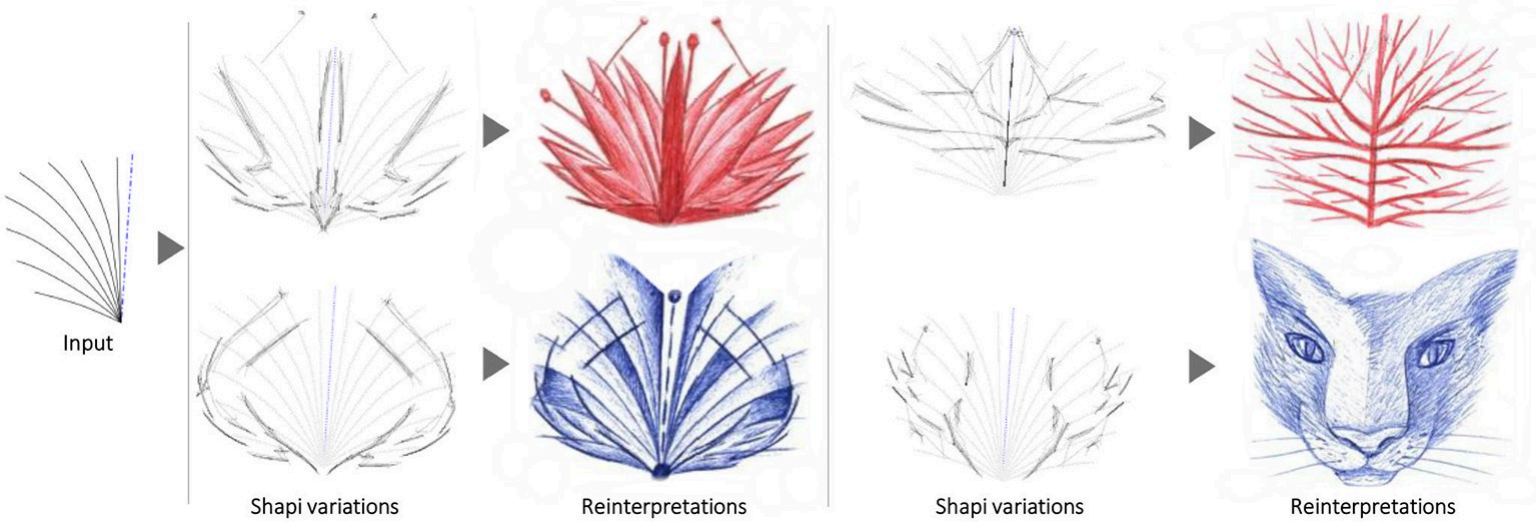
Some implementations with artistic open-ended purposes. The Seeds of three objects (hand-axe, lamp shade, and bottle) are used to reinterpret and discover new entities (human sketches on the right).

**Fig 18:**
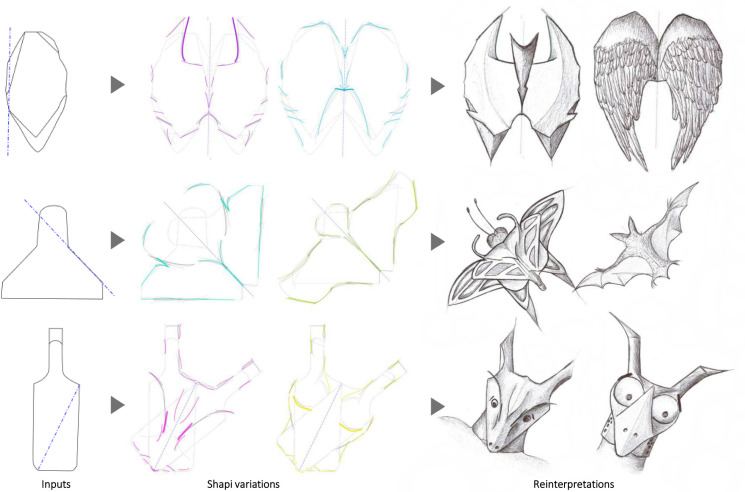
Using an abstract Seed to generate new emerging patterns that inspire divergent human interpretations.

Similar to Sec. 5.2, the strategy to increase divergence involves using symmetry axes in unconventional positions and aggressive settings for the Nudge parameters. The Seeds lead to the reinterpretation and discovery of new objects and creatures: from a car silhouette to aerospace vehicles (Fig 6 middle), from a perfume bottle to cartoon faces (Fig 6 bottom), from a hand-axe to an abstract bat and angel wings (Fig 17 top), from a lamp shade to a butterfly and a bat (Fig 17 middle), and from a bottle to cartoon creatures (Fig 17 bottom). Similarly, some shape variations taken from Fig 15 are reinterpreted with more open-ended goals in Fig 18.

## 6. Survey on perceived value

A survey is developed to evaluate the perceived value of Shapi’s capabilities represented in shape variations from the case studies (Sec. 5). The original Survey and the complete sheets of results can be found at [33]. This work aims at assessing whether a tool like Shapi and its general approach could be perceived as potentially valuable to the academic and practicing design communities. Questions about usability, extensive evaluations of the specificities of the tool, or interface design are beyond the current scope (see Sec. 3.3).

**Fig 19:**
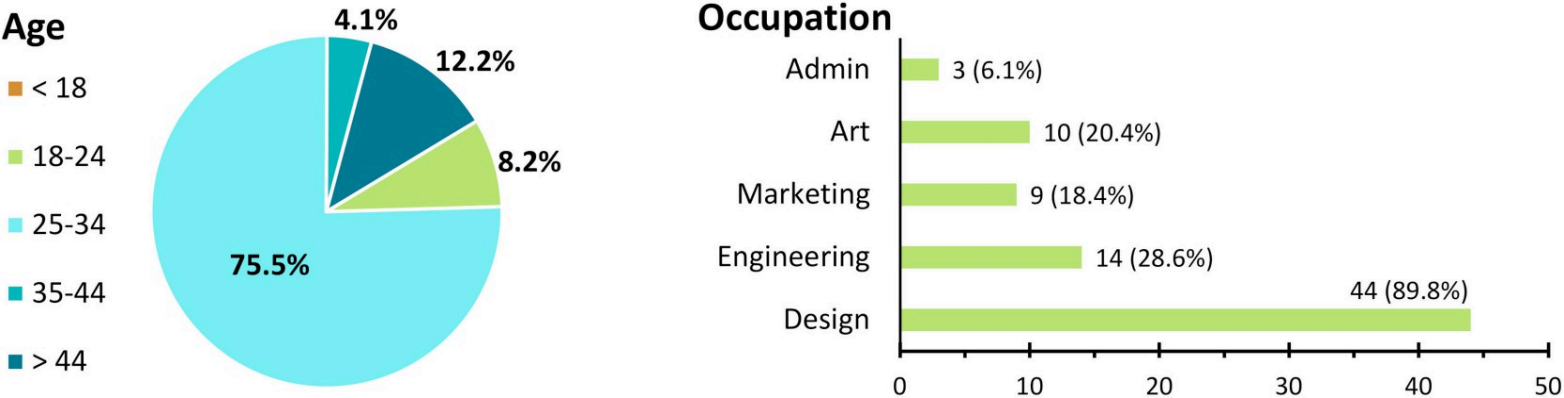
Survey respondents by age and field of occupation. Respondents may choose more than one occupation.

49 people responded to the survey (Fig 19), with most of them having an occupation related to design (almost 90%) and art (20.4%). Their majors include Product Design Engineering, Mechanical Engineering, Industrial Design, Graphic Design, Fashion Design, Architecture, Marketing, Advertising, and Art. The options regarding background and occupation are not mutually exclusive. For example, a participant may have an occupation related to both design and marketing, or an administrative occupation but with a background in design. Also, design can be multidisciplinary, with marketing and design departments working closely and marketing or administrative team members influencing the decision process on which tools are acquired and used. 67.3% of them have postgraduate studies, and most of them are design students, design graduates, entrepreneurs, and at least 11 of them have taught university-level design courses.

**Fig 20:**
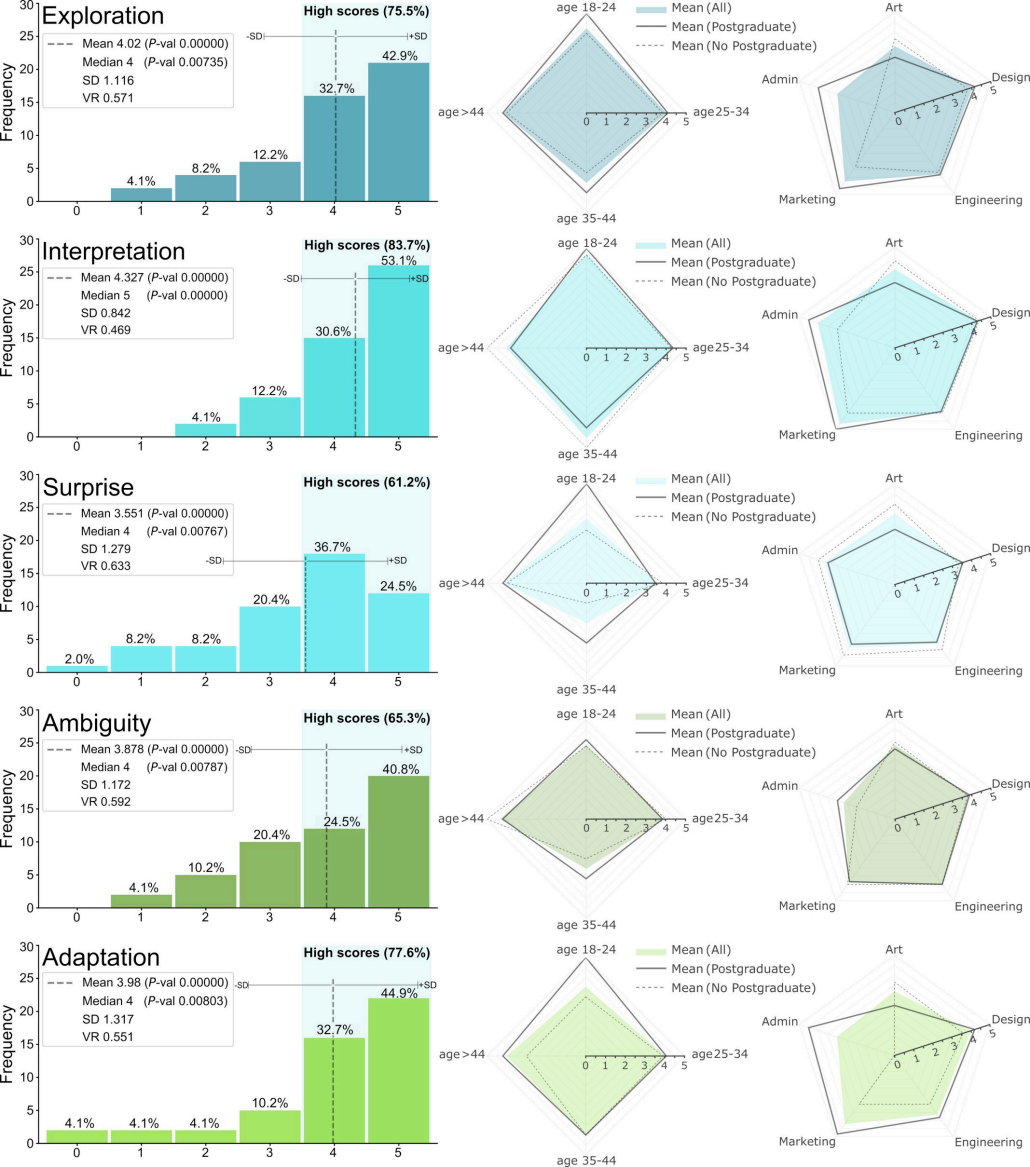
Survey results of perceptions about Shapi’s capabilities and potential value with 0–5 scores. Results are displayed by frequency, age group, occupation, and Postgraduate studies. Metrics include mean, median, Standard Deviation, Variation Ratio, and *P*-value.

**Fig 21:**
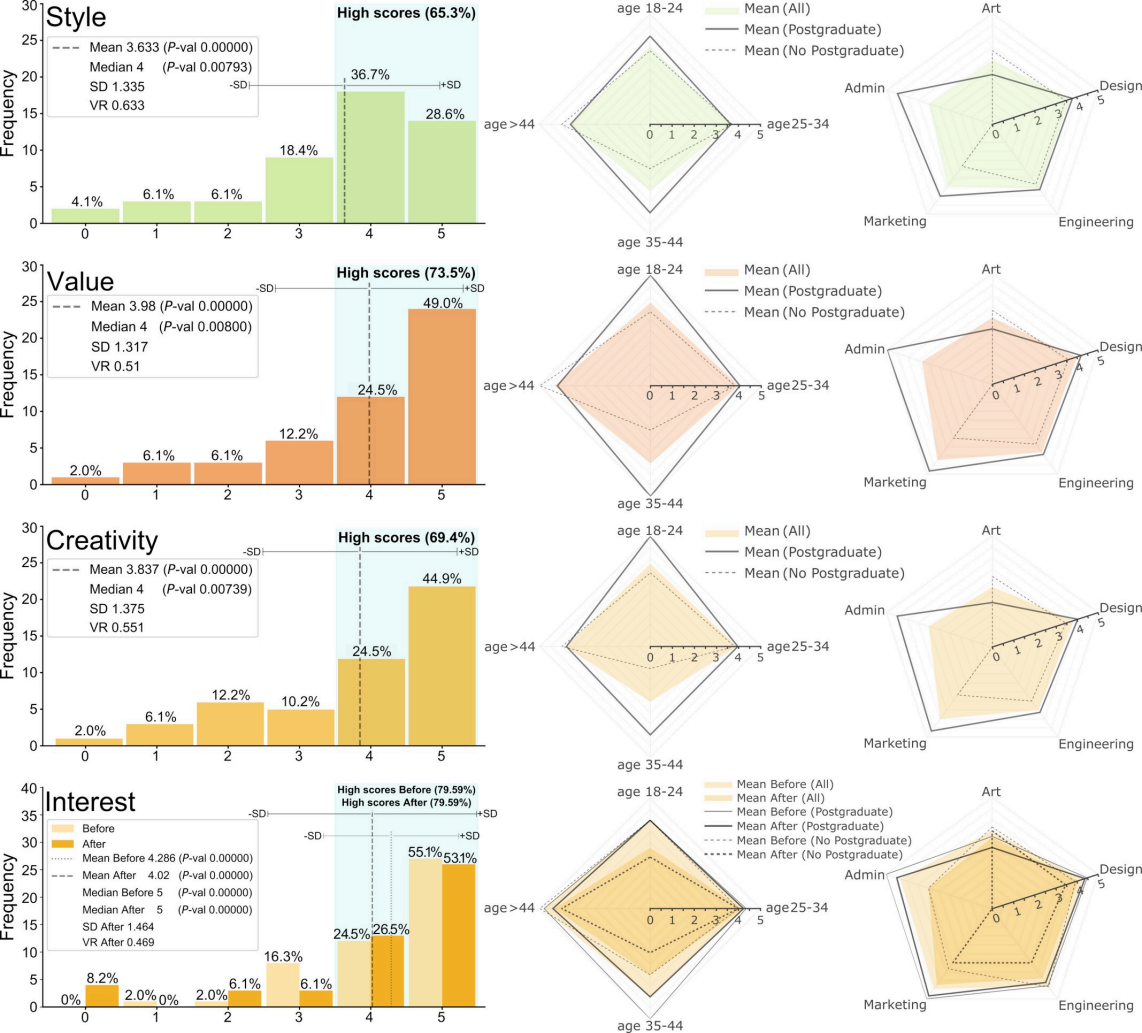
Survey results of perceptions about Shapi’s capabilities and potential value with 0–5 scores. Results are displayed by frequency, age group, occupation, and Postgraduate studies.

Figs 20 and 21 present the distributions of scores for the constructs (a-h) (see Sec. 3.3), as well as the Interest Before and After being exposed to the demonstrative cases. Defining ‘high rating’ as a score ≥4 out of 5, the perception assessment results in a majority of high scores ranging from 61.2% (for Surprise) to 83.7% (for Interpretation). The mean values range from 3.551 (Surprise) to 4.327 (Interpretation), the modes from 4 (Surprise and Style) to 5 (all others), and the medians from 4 to 5 (only Interpretation and Interest). Thus, Surprise (i.e., are there non-obvious variations?) is consistently among the lowest rated perceptions and Interpretation (i.e., the variations preserve the essence) among the highest. This is an interesting trade-off because generating shape variations that notably depart from the obvious while at the same time preserve the original essence is challenging. Also, both SD and VR are the lowest for Interpretation, suggesting that there is more consensus (less dispersion) regarding this perception. The perceptions with the most dispersion are Interest, in terms of SD, and Style and Surprise, in terms of VR. All means and medians are statistically significant with *P*-values < 0.01. The results suggests that the score distributions are unlikely to be originated from randomness alone (Null hypothesis: scores are drawn from a discrete uniform distribution).

Although the sample of respondents may not necessarily be representative of the entire population of potential users and the different classifications of age, occupation, and studies are not uniformly distributed, the differences lead to interesting exploratory insights. For Exploration, scores are lower for age 35–44, Art and Administrative occupations, while Marketing is higher and having Postgraduate studies leads to higher scores except in Art. For Interpretation, scores are lower for age > 44 and Art, while Marketing and Design are higher and Postgraduate studies lead to positive or moderate effects except in Art. For Surprise, scores are lower for ages 18–24 and 35–44 and very similar by occupation, including Postgraduate studies having a moderately negative effect except in Design. The trends for Ambiguity are similar to those for Surprise, except for Administrative being considerably lower. For Adaptation, scores are lower for age 18–24, Art, and Administrative, while Postgraduate studies have a considerably positive difference except in Art. For Style, it is lower for age 35–44, Art and Administrative, while Postgraduate studies relate to higher scores except in Art and age > 44.

Value and Creativity are perhaps the most general and directly relevant perceptions regarding the purpose of this work (see Sec. 3.3). Both are lower for age 35–44 and Art, higher for Administrative and Marketing, and Postgraduate studies relate to higher scores except in Art and age > 44. While the level of Interest is highly conditioned on the motives of each respondent, it may be used as a proxy for the general perceived desirability of a tool like Shapi, and the before-after comparison as a proxy for expectations being met (or not). Interest is lower and dropped more for ages 18–24 and 35–44, it is lowest in Art and dropped the most in Administrative occupations. The proportions of high scores for Interest, both before and after, remain at almost 80%. The differences between the Interest distributions before and after are not statistically significant, with *P*-value 0.33792 for means and *P*-value 1.0 for medians. Thus, the null hypothesis appears to be likely in this case (i.e., both groups may come from the same distribution and differences in means and medians are likely to be due to randomness). This can be explained by the possibility that initial expectations (based on a brief description of the tool’s capabilities) are in good agreement with the demonstration cases.

**Fig 22:**
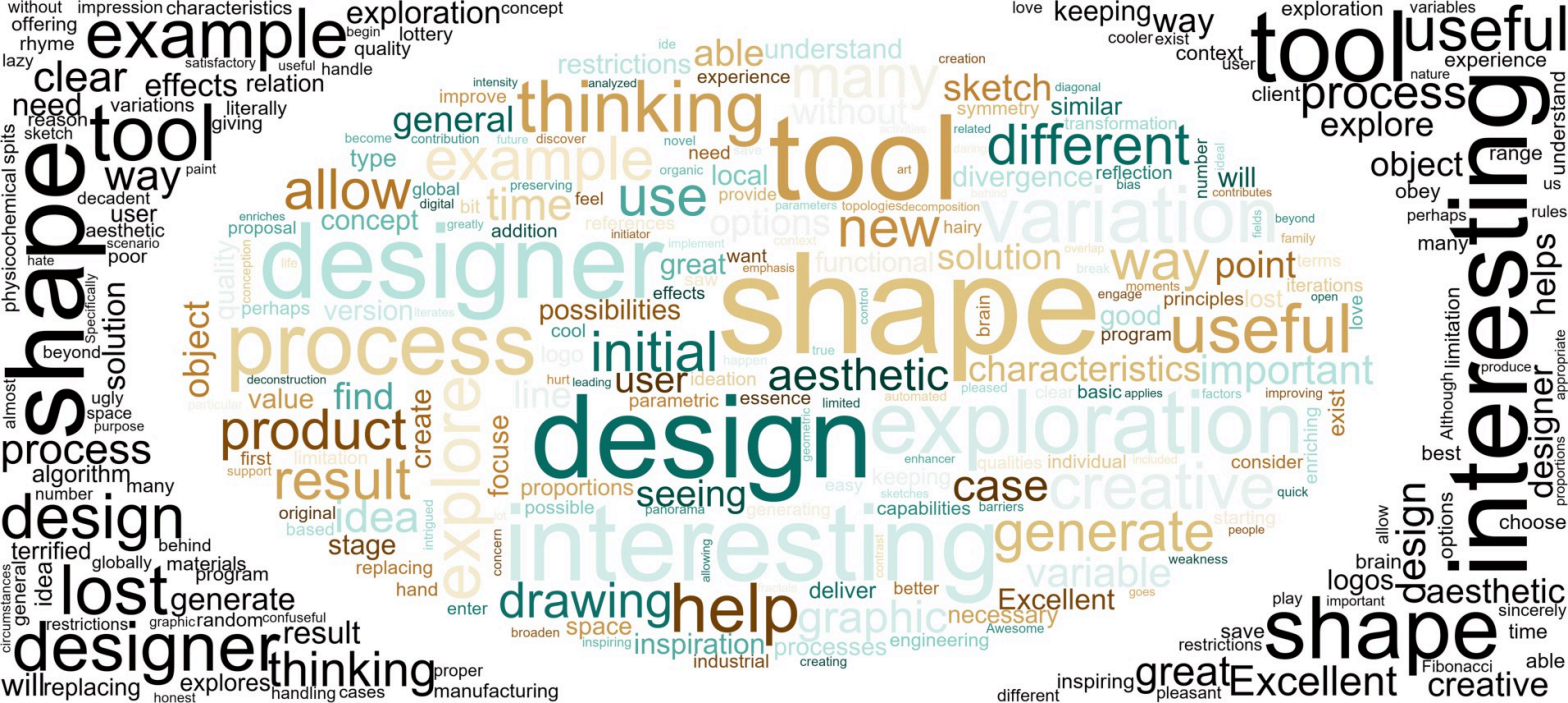
Word Clouds produced with the open feedback from the survey using all sentences (center), only the 20 most negative sentences (left), and the 20 most positive (right).

96% of respondents provided open feedback at the end of the survey, which is used in this work as a valuable space for gaining insight about general impressions, recommendations, paths forward, and concerns. This feedback is graphically analyzed and summarized in Fig 22 (center) by identifying and highlighting a hierarchy of words based on frequency and salience, such as design, exploration, interesting, and thinking. Sentiment analysis is implemented to estimate the Polarity of every feedback sentence, ranging from -1 (most negative) to 1 (most positive). Based on Polarity, the 20 most negative and 20 most positive sentences are identified and analyzed in the form of word clouds (Fig 22 left and right), with salient words like lost, terrified, replacing, lazy, decadent, poor, and ugly on the negative side, and interesting, excellent, creative, useful, great, understand, aesthetic, and inspiring on the positive side.

**Fig 23:**
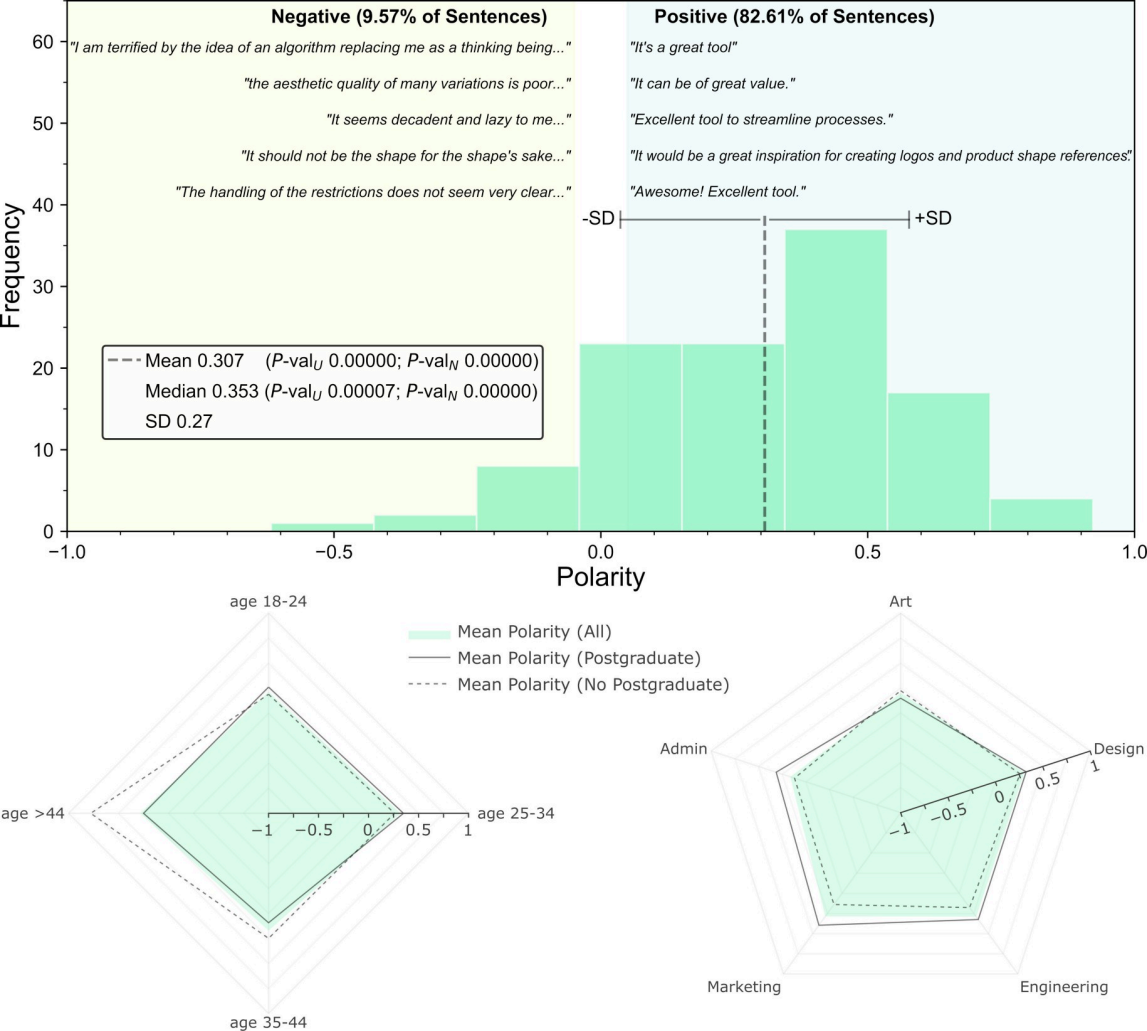
Polarity of the open feedback from the survey. It is displayed by frequency, age group, occupation, and Postgraduate studies.

Fig 23 shows the 5 most extreme sentences (in both directions of Polarity) and the Polarity distribution, which is fairly uniform across occupations and age groups. Some of the open feedback is critical of the tool and some participants are worried about the prospect of algorithms replacing designers (Sec. 7.1), but most of the feedback (>80%) appears to be supportive and is classified as being positive, i.e., Polarity > 0.05. The mean and median are both highly statistically significant, with *P*-values < 0.0001 using a continuous uniform distribution (*P*-val_U_) or a normal distribution (*P*-val_N_) for the null hypothesis. Therefore, the Polarity scores are unlikely to be originated from randomness alone.

**Fig 24:**
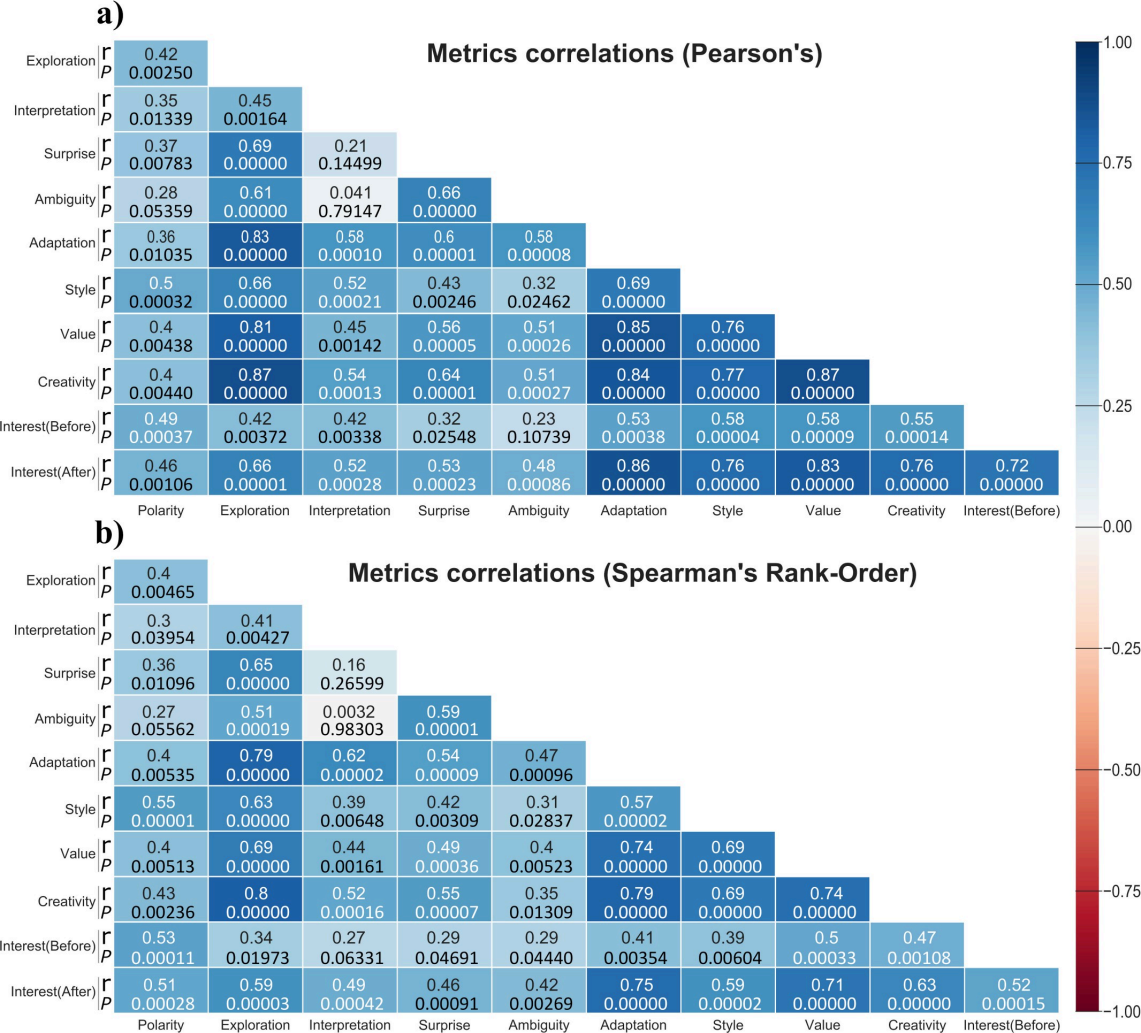
Correlations (r) and their *P*-values (*P*) comparing all distributions of perception scores and polarity.

The correlations between all distributions of perception scores and Polarity are presented in Fig 24. All are positively correlated, except for Ambiguity and Interpretation (almost neutral). Overall, Pearson’s and Spearman’s correlations (r) appear to describe similar relationships. Polarity is most related to Style and Interest; Exploration is most related to Creativity, Adaptation, and Value; Interpretation is most related to Adaptation; Surprise is most related to Exploration, Ambiguity, and Creativity; Ambiguity is most related to Surprise and Exploration; Adaptation is most related to Interest (after), Value, and Creativity; Style is most related to Creativity, Value, and Interest (after); Value is most related to Creativity, Adaptation, and Interest (after); Creativity is most related to Exploration, Value, and Adaptation; Interest (before) is most related to Interest (after); and Interest (after) is most related to Adaptation, Value, Creativity, and Style.

With a 5% significance threshold, most correlations are statistically significant except for Ambiguity-Polarity, Surprise-Interpretation, Ambiguity-Interpretation, Interest (before)-Ambiguity, and Interest (before)-Interpretation. The strongest Pearson’s correlations (≥0.85) are Creativity-Exploration, Creativity-Value, Adaptation-Interest (after), and Adaptation-Value. The strongest Spearman’s correlations (≥0.79) are Creativity-Exploration, Creativity-Adaptation, and Adaptation-Exploration. As discussed in Sec. 3.3, it is worth noting that the constructs of Value and Creativity are more general and are not completely independent from other constructs like Exploration, Interpretation, Adaptation, Style, and Surprise. These relationships at the construct level seem to be in agreement with the resulting correlations, which are relatively strong and statistically significant in the case of the abovementioned variables. Furthermore, all Interest correlations (except Polarity) increase from before to after, suggesting that shifts in Interest may be related at least in part to those other perceptions.

## 7. Discussion

This section contextualizes the purpose and contributions of this work, describes the main insights (Sec. 7.1), and discusses the limitations and paths forward (Sec. 7.2). Insights are classified as positive, negative, risks, or opportunities, which will hopefully be a valuable contribution for researchers developing similar tools, the methodologies around them, and the potential practical implementations toward human-AI collaborations in shape exploration. Despite the many merits of the interesting tools that have already been developed, it appears from the literature review that the problem of assisting early exploration of shapes with computational tools has not been fully resolved yet, which is exemplified by designers still commonly favoring freehand sketching and deferring the use of computational tools to later stages (Secs. 1–2).

Shapi is one attempt at developing a tool that addresses some of the pitfalls that have been reported in the literature. To be less disruptive of the ‘natural’ workflow, the means to communicate with the tool is trough freehand sketching and observation. The user provides a sketched Seed, reflects on the shape variations offered as a response, and then freely reinterprets the shape for future human-AI cycles of exploration, or for moving toward more refined details in parallel or successive cycles. The user can interactively control the level of divergence, but the role of the tool is mostly as an autonomous assistant, without forcing the user to engage too early with low-level geometric manipulations constrained to a digital platform with limited features (as with some CAD tools), or requiring extensive fine-tunning, dataset building, and model training (as with many machine learning tools). Shapi is designed to embrace early shape ambiguity in the inputs and outputs, without forcing the user to detail the shapes more than desired or providing ready-made or complete solutions, which has been reported to lead to premature convergence. As opposed to replacing human sketches, Shapi’s variations are meant to be intermediary What-If scenarios, cognitive facilitators to deepen shape understanding and catalyze ideas. Hopefully, these variations may help overcome creative blocks and design fixation [22] with a stream of relevant visual stimuli [94]. Shapi as a proposed tool and workflow could be evaluated in many ways, but the main focus of this work is to assess the perceived value of the capabilities of such a tool.

### 7.1 Main insights

#### The positive

- Representing simple shapes in vector form with an evocative degree of ambiguity, incompleteness, and roughness: The solution proposed to address this technical challenge involves spatially grouping the contour points with K-Means Clustering; identifying high-interest points based on measuring proportional point-density variation with a Finite-Difference scheme; partially fitting cubic Bézier curves with a Genetic Algorithm; superimposing groups of resulting phenotypes; and applying selective emphasis of color and line thickness. Based on the 17 case studies explored, this seems to be a promising approach that is adaptable enough to represent a wide variety of shapes (Mean Adaptation 3.98, 77.6% high scores), with a meaningful degree of Ambiguity (Mean 3.88, 65.3% high scores), and a relatively appealing Style (Mean 3.63, 65.3% high scores) without losing the essence of the Seed (Mean Interpretation 4.33, 83.7% high scores). The resulting style appears to resemble rough human line drawings with charcoal or pencil, which can be valuable as potentially evocative thinking sketches (not as final presentation sketches).
- Local and global shape variations with an interactive control of stochasticity without losing the essence of the Seed: The solution proposed to address this technical challenge is the ‘Nudge’ function (aided by drawn Boundaries and Symmetry axes), which can be controlled in aggressiveness and locality by varying the mean deformation force, radius of influence, frequency, and the region where a Nudge center can occur. This approach seems promising for the purpose of generating variations within a delimited solution space (Mean Exploration 4.02, 75.5% high scores) with some non-obvious variations (Mean Surprise 3.55, 61.2% high scores). As stated by one participant, ‘I was very surprised by the variations because they look a lot like what a designer does in the design process’.
- Perceived value of the tool’s capabilities within the proposed workflow: Based on the survey’s scores and open feedback, there is a mostly positive and open-minded perception for a tool with Shapi’s capabilities, at least within this work’s limitations. All metrics have a majority (>60%) of high scores and most (>80%) open feedback sentences are classified as positive, with the highest in Polarity underscoring concepts like interesting, creative, useful, understand, aesthetic, and inspiring. The most numerous age groups are also the ones with the most favorable perceptions of such a tool, with ages 25–34 (75.5%) likely corresponding to practitioners who recently completed or are close to completing their education, and ages >44 (12.2%) likely corresponding to design Professors and more experienced practitioners. Having postgraduate studies is also related to more favorable perceptions in most metrics, perhaps due to higher exposure to design tools and alternative methodologies. The proportions of high scores for Interest, both before and after, remain at almost 80%, and the before-after distributions are not significantly different (*P*-value 0.34–1.0). This may suggest that the expectations that many of the respondents developed while reading the description of the tool were in agreement with the demonstrations that were shown to them.

#### The negative

- Representing more complex shapes: The proposed approach for representing shapes as loose sketches appears to diminish in evocative effectiveness and consistency as the shape complexity increases. For instance, the car silhouette exploration seems to be considerably less compelling than the liquor bottle exploration (simpler and symmetric Seed). This can be partially mitigated by increasing the number of Bézier curves and K clusters but only to a limited degree and at higher computational cost. This may lead to negative perceptions of insufficient value, e.g., ‘…not only with simple silhouettes, but also with more complex shapes’.
- The style may be unappealing for some: Although the purpose of Shapi’s outputs is not to generate final sketches, it is important that shape variations are perceived as aesthetically appealing and evocative because that serves as motivation for continuing the sketching conversation. Some participants reported that such a tool should ‘generate shapes with slightly sharper lines’ and that ‘the line looks discontinuous and hairy’. This is a difficult trade-off to navigate, as sharper shapes tend to be more aesthetically pleasing but also less ambiguous, which is one of the key aspects to avoid idea fixation and premature convergence.
- Divergence was not surprising enough for some: Although Exploration is among the highest-scoring perceptions, Surprise is the lowest (Mean 3.551, 61.2% high scores). Some participants state that such a tool ‘Must be more daring’ and that ‘…it doesn’t give me many creative variations’ or ‘…it is still predictable’. There appears to be a challenging trade-off because setting Shapi or a similar tool to generate more surprising variations also risks generating more unreasonable variations (e.g., an unstable base or a bottle-neck that is too narrow) that lose the essence of the Seed (e.g., something that does not even look like the same type of product).

#### The risks

- Perceptions of young students: It would seem that one of the clearest potentials for such a tool would be as an educational resource to assist young design students. However, somewhat paradoxically, the youngest age group surveyed (Age 18–24) appears to have some of the least favorable perceptions, i.e., comparatively lower scores and a considerable drop in Interest. If similar tools were to be proposed with educational purposes, this is a risk that deserves further inspection.
- Misunderstood purpose: There seems to be a considerable risk for such a tool to be misunderstood in purpose, i.e., getting the impression that the whole shape exploration would migrate to a limited digital platform or would be delegated to an AI, that all variations would be random and uncontrollable, or that design would be reduced to looking for appealing shapes. Although those are not the views held in this work (Sec. 4.1), it appears that some participants got that impression, e.g., ‘It should not be the shape for the shape’s sake’.
- Algorithm aversion: A risky scenario with human-AI interactions is algorithm aversion, i.e., when potential users develop a negative bias against being assisted by algorithms and ignore recommendations in favor of retaining control even if performance gains are knowingly being sacrificed [95]. In other fields where human-AI tools have been faced with resistance, such behaviors have been reported to emerge from fears of being replaced, deskilling, and devaluation of experiential knowledge [96]. As one participant states in the survey, ‘I am terrified by the idea of ​​an algorithm replacing me as a thinking being… they would make design an obsolete profession. It seems decadent and lazy to me’. This work emphasizes the role of a tool like Shapi as an assistant and collaborator as opposed to a replacement.
- Automation bias: Automation bias [95] is when users blindly rely on the AI recommendations in detriment to their own role in the interaction. Some participants were apparently worried about this risk with a tool like Shapi, e.g., ‘I just have a concern about the true contribution of the designer’ and ‘it can also limit freedom of expression… It is important to complement it with drawing techniques and that it is assumed as an additional help’.

#### The opportunities

- Taking advantage of ambiguity: Ambiguity and Interpretation have an almost neutral correlation (r 0.04), which may suggest that some degree of ambiguity can be exploited in the variations while still maintaining the essence of the Seed (high Interpretation). Ambiguity is correlated the most with Exploration and Surprise, which presents an opportunity to improve the metric with the lowest score (Mean Surprise 3.55) by exploring Ambiguity further.
- Improved Adaptation and Style: Interest (after) is highly correlated to Adaptation (r 0.86) and Style (r 0.76), so perhaps improving the adaptability (e.g., being capable of dealing with a broader range of shapes) and aesthetic appeal of the outputs produced by a tool like Shapi would be effective toward increased Interest.

- New functionalities: Several participants propose additional functionalities that could be used for a richer control of the stochastic variations, e.g., enforcing design principles like overlap, diagonal, contrast and emphasis, or enforcing proportions based on fractals or the Golden ratio. Some propose more direct ways of interacting with the variations, such as turning them into editable meshes or allowing the selection and blending of different outputs. This could be developed as an additional functionality to allow for greater control or reduced rework by generating outputs that could be directly used in the next design phases. But the main focus would still be human reinterpretations in the form of sketches as opposed to direct low-level geometric manipulations, which has been reported to lead to fixation and excessive distractions (Sec. 1). Additionally, one participant proposes that such a tool should be made into a plug-in for Adobe softwares as opposed to an additional software, which would be an interesting approach toward being less disruptive of the ‘normal’ workflow in the case of digital art.
- Marketing: The participants with occupations involving marketing score most metrics comparatively higher. Perhaps these participants perceive the potential value of such a tool in the market, or perhaps they are more attuned with current trends regarding how AI and digital tools are transforming many industries.
- Area of Application: It appears that art-making is too open-ended to be fully supported by a tool like Shapi, which is reflected in the observation that art-related participants scored key metrics comparatively lower (Value, Creativity, and Interest). Graphic design seems like a promising area of application as long as a tool like this integrates seamlessly with other popular digital tools. It seems that industrial design is where a tool like Shapi would be the most valuable, which is in agreement with consistently high scores on the part of designers. As with the car case, emergent patterns that could be interpreted as emerging product features (e.g., headlights) could be developed further in human reinterpretations. Also, it seems that the automatically detected shape regions lead to meaningful local explorations, e.g., bottle (cap, neck, main body) or axe (point, palm support, index finger surface). The variations seem to be particularly effective for simple and symmetric products, as the shape can be represented more consistently and enforced symmetry adds some structure to the stochastic deformations.
- Purpose: Based on the open feedback, a tool like Shapi could be perceived as a valuable educational resource, e.g., ‘it is a very useful tool for students in the first semesters, since this tool highlights the processes of geometrization and the principles of shape transformation’ and ‘It can also greatly help people who say “I am not creative” as an enhancer or initiator of a creative process’. For professional practice, some stated purposes include ‘ideal to generate new shapes’, ‘An excellent way to save time and explore ways that you might not be able to experience due to your brain’s own limitation’, ‘to expand the solutions space’, and ‘for moments of quick ideation. Or to create a family of products’.

### 7.2 Limitations and future work

It is worth stressing that generating a design concept is by no means only a matter of free shape exploration. There are many aspects beyond an appealing shape, such as functionality, usability, and manufacturing constraints and considerations [5,69]. Nonetheless, the design process is comprised of various stages that can go in parallel or in series, where a product concept is made progressively more detailed, complex, and realistic. This work concentrates only on the early conceptual phase [15], where there can usually be a relatively free shape exploration focused mainly on the general appearance [14], which can then be made more constrained and sophisticated in the following phases.

One important limitation of the technical approach is that it only seems to work well with abstract shapes or 2D side-views. Nonetheless, sketching in 2D is reported to still play a key role in early shape exploration, as it is commonly faster and easier [97] and helps reduce cognitive overload [43]. Some examples of product shape explorations that partially rely on side-views include concepts about cars [48], kettles [98], and mugs, hair dryers, bottles, mice, and helmets [99]. However, as displayed in Fig 1, Shapi variations are intended to inspire and be reinterpreted as free human sketches, which can themselves be 3D (e.g., in perspective). If Shapi’s capabilities are expanded so that 3D representations can be effectively manipulated, this will likely require some kind of autonomous semantic understanding of the distinct abstract volumes. This is another limitation of the tool, as divergence is not generated based on sematic understanding, but on partially fitted curves, unsupervised clustering based on geometrical proximity, and interactively controlled stochastic Nudges. Thus, some of the variations are unfeasible as concepts, such as a jar without an opening or a handle that is too small, although such outputs can still inspire feasible ideas in human reinterpretations.

The survey was answered by a set of highly relevant participants including design professors, practitioners, and entrepreneurs, and the survey’s purpose was focused on depth more than quantity. The participants scored the various metrics and provided open feedback based on their expertise and their perceived value of the tool after being exposed to a brief description of the workflow and a wide range of demonstrations. For future work, it would be valuable to have a larger group of respondents that is more uniformly representative across various factors like age, occupation, and expertise level. It would also be valuable to evaluate the usability of the tool with a working software application that includes a user interface, and compare the results against a control group.

Based on the survey insights (Sec. 7.1), there are new functionalities about controlled divergence that seem promising for future work. Moreover, additional user control could be considered by developing a way to establish which particular region to focus the exploration on and delimiting a region that should remain unchanged. Lastly, a secondary AI algorithm could be implemented to select the variations that are more likely to be perceived as aesthetically interesting before presenting them to the user.

## 8. Conclusions

Shape divergence can be challenging and time-consuming, especially for novices, which is why there has been considerable research interest in developing computational tools for this purpose. In an effort to try to address some of the common pitfalls reported in the literature, a new tool (*Shapi*) is proposed for assisting early shape exploration. Its capabilities are explored in a diverse set of implementation case studies and an exploratory assessment of perceived value is conducted. While the human creator controls the overall shape exploration, Shapi is meant to be an assistant that facilitates zooming in and out of the process by providing local and global shape variations as intermediary What-If scenarios and cognitive facilitators. The intention is that when the creator is stimulated with variations of an initial shape, it becomes easier to understand the shape better, identify what works and what doesn’t, and discover potential interesting directions. To avoid premature convergence, the approach embraces ambiguity as a central part of early divergence.

The approach proposed for representing shapes in vector form seems to be promising in terms of Adaptability (77.6% high scores), meaningful Ambiguity (65.3% high scores), the rough Style (65.3% high scores), and the capability to maintain the essence of the input sketch (83.7% high scores in Interpretation). The proposed Nudge function appears to be an effective divergence mechanism for Exploring shapes within a delimited solution space (75.5% high scores) with some non-obvious variations (61.2% high scores in Surprise). A participant underscored the similarity of these autonomous variations to those made sometimes by designers.

One of the main risks identified is for the tool’s purpose to be misunderstood, potentially leading to algorithmic aversion due to fears of being replaced, deskilling, and devaluation of experiential knowledge or, on the other hand, leading to automation bias due to an overreliance on the tool’s suggestions. Nonetheless, there was a mostly positive and open-minded perception of the approach based on the survey’s scores (all perception aspects had > 60% high scores) and the open feedback (>80% classified as positive). Moreover, the results suggest that the expectations that most of the respondents developed while reading the description of the tool agreed with the example outputs from case studies (Interest before and after remained with almost 80% high scores).

Regarding potential applications, the tool was envisioned to be particularly useful during the early conceptual stages in Industrial Design. Perhaps to a lesser degree, the capability to explore abstract patterns may also be useful to support Graphic Design and open-ended art projects. Based on the open feedback, the perceived potential purposes for this tool include overcoming creative blocks, time-saving, generating families of products, or as an educational resource. As a whole, this work can bring insight into the intersection and collaboration between artificial intelligence, design, and art, including some promising functionalities, potentially constraining limitations, risks, and opportunities. The results appear to be encouraging of the general focus, namely, a tool that converses with the user through shape variations in the form of rough sketches, with some degree of interactive control, and that generates evocative intermediate suggestions that enable a deeper understanding and inspire interesting directions.

## Supporting information

S1 Fig Sketch representation. a) Clustering and characterization of symmetry and boundary points, b) identification of high-interest points, c) initialization of cubic Bézier curves, d) RMSE fitness function, e) genetic mutations, and f) genetic cross-over. In a fixed number of generations, the GA creates a large population of sets of curves that are partially fitted to the contour points. Among the entire population across all the generations, a Roulette Wheel scheme based on RMSE fitness is used to select groups of 10 individuals. By fusing (superimposing) their phenotypes, the style can resemble rough and loose sketches where the contour is discontinuous and somewhat ambiguous. Additionally, to make the sketches more evocative, stochastic selective emphasis is used to highlight with color and a greater thickness a particular curve per cluster.
(TIF)

S2 Fig Nudge function proposed to generate greater diversity. It can be used to implement stochastic local or global contour deformations by displacing the Bézier control points within the radius of influence (R).
(TIF)

S1 Table Most relevant input parameters for controlling the Shapi algorithm. Tentative Default values are suggested. The inputs with high interactive relevance are identified with ’*’. Table S1 presents some suggested default values for the algorithm’s parameters. Identifying the optimal values for a given input sketch and a given exploration purpose is a considerably complex and time-consuming task. Thus, it is suggested for the user to start with the default values and then try slight deviations in the parameters marked with (***) or (**). These default values are found to work reasonably well after running the Shapi algorithm with hundreds of different configurations and considerably different kinds of shapes (e.g., from a hand-axe to a car silhouette), and with input images ranging from 110*170 to 344*127 pixels. The size of the input image is relevant because several parameters are set in pixel units.
(DOCX)

S1 Appendix (DOCX)

S2 Appendix (DOCX)

S3 Appendix (DOCX)
