# Supplementary figures and images for: The perceived value of human-AI collaboration in early shape exploration: An exploratory assessment
Source: PLoS One. 2022 Sep 12;17(9):e0274496. doi: 10.1371/journal.pone.0274496 (PMC9467378; doi:10.1371/journal.pone.0274496)

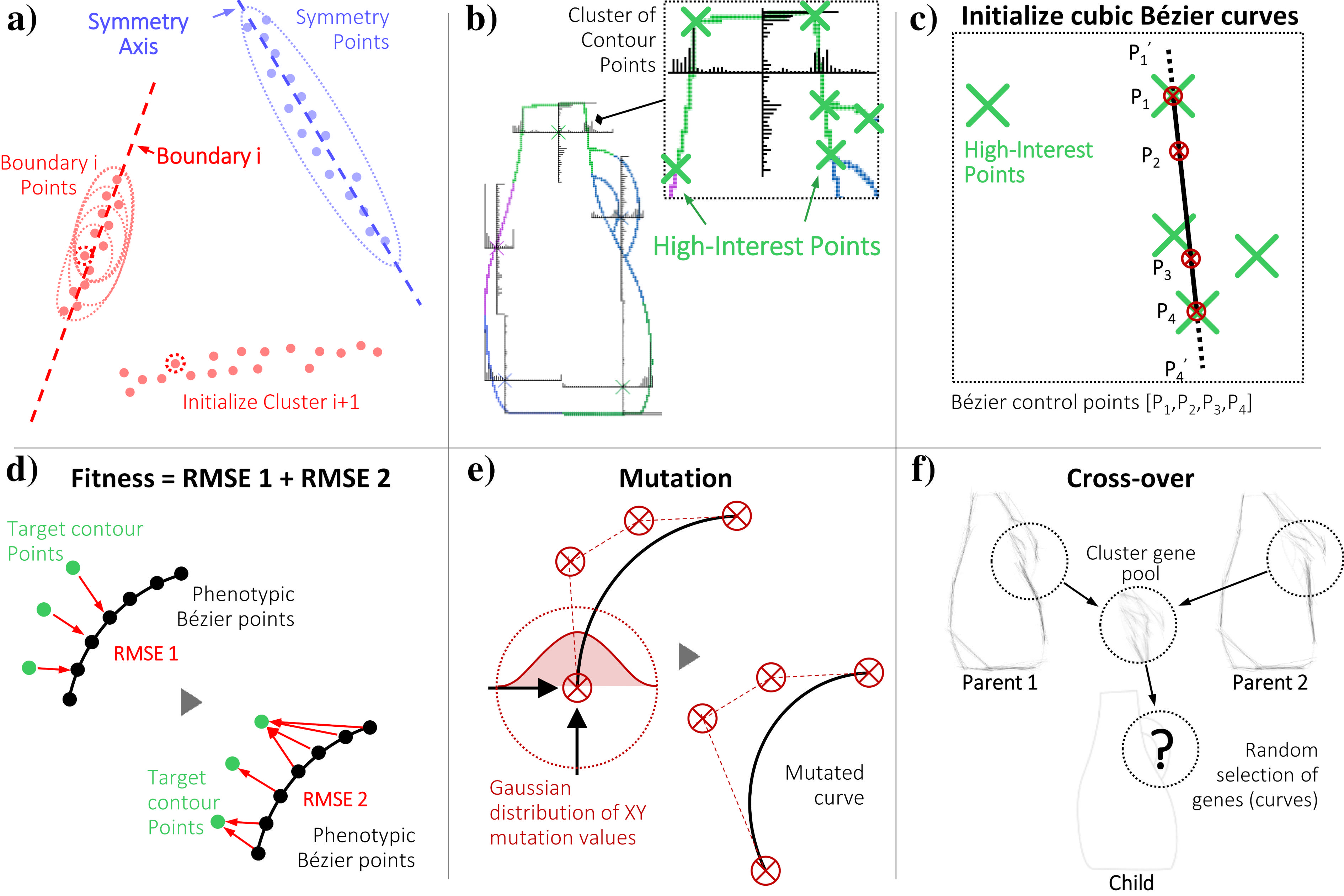

Supplement: S1 Fig — a) Clustering and characterization of symmetry and boundary points, b) identification of high-interest points, c) initialization of cubic Bézier curves, d) RMSE fitness function, e) genetic mutations, and f) genetic cross-over. In a fixed number of generations, the GA creates a large population of sets of curves that are partially fitted to the contour points. Among the entire population across all the generations, a Roulette Wheel scheme based on RMSE fitness is used to select groups of 10 individuals. By fusing (superimposing) their phenotypes, the style can resemble rough and loose sketches where the contour is discontinuous and somewhat ambiguous. Additionally, to make the sketches more evocative, stochastic selective emphasis is used to highlight with color and a greater thickness a particular curve per cluster. (TIF) [file pone.0274496.s001.tif]

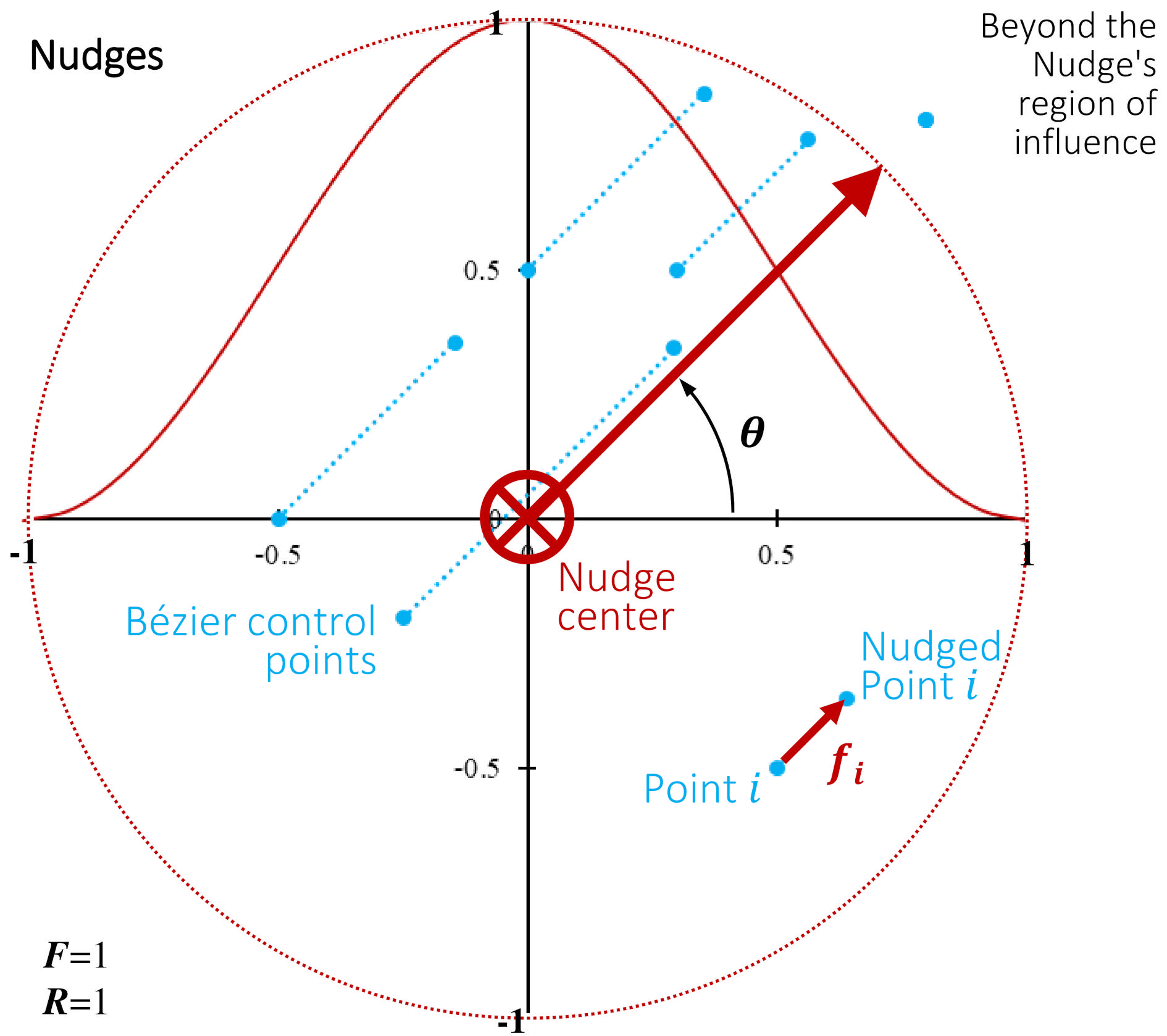

Supplement: S2 Fig — It can be used to implement stochastic local or global contour deformations by displacing the Bézier control points within the radius of influence (R). (TIF) [file pone.0274496.s002.tif]
