# Supplementary material for: The perceived value of human-AI collaboration in early shape exploration: An exploratory assessment
Source: PLoS One. 2022 Sep 12;17(9):e0274496. doi: 10.1371/journal.pone.0274496 (PMC9467378; doi:10.1371/journal.pone.0274496)
Supplement: S1 Table — Tentative Default values are suggested. The inputs with high interactive relevance are identified with ’*’. Table S1 presents some suggested default values for the algorithm’s parameters. Identifying the optimal values for a given input sketch and a given exploration purpose is a considerably complex and time-consuming task. Thus, it is suggested for the user to start with the default values and then try slight deviations in the parameters marked with (***) or (**). These default values are found to work reasonably well after running the Shapi algorithm with hundreds of different configurations and considerably different kinds of shapes (e.g., from a hand-axe to a car silhouette), and with input images ranging from 110*170 to 344*127 pixels. The size of the input image is relevant because several parameters are set in pixel units. (DOCX) [file pone.0274496.s003.docx]

**Table S1. Most relevant input parameters for controlling the Shapi algorithm.**

| Groups | Input Parameters | Defaults |  |
| --- | --- | --- | --- |
| Point classification | Saturation Tolerance [0,255] | 210 |  |
|  | No. Contour clusters | 10-14 | *** |
|  | No. K-Means Clustering iterations | 50 |  |
|  | Proportional XY point-density variation Tolerance | 0.55 |  |
| Bézier curves | No. Bézier curves per phenotype | 25 | *** |
|  | No. Bézier phenotypic points per curve | 5 |  |
|  | Initialized curve extrapolation [Proportion] | 0.1 |  |
| Genetic Algorithm functions | RMSE search square width [Pixels] | 5 |  |
|  | No. individuals per GA generation | 50 |  |
|  | Proportion of Elites | 0.5 |  |
|  | No. Generations | 20 |  |
|  | Mutation Probability | 0.25 |  |
|  | Mutation Magnitude Standard Deviation [Pixels] | 2 |  |
| Nudges | No. Global Nudges per Global shape variation | 1-2 | ** |
|  | No. Local Nudges per Local shape variation | 1-2 | ** |
|  | Mean Global Nudge Force ($\bar{F}$) [Pixels] | 15 | ** |
|  | Mean Local Nudge Force ($\bar{F}$) [Pixels] | 10 | ** |
|  | Global Nudge Standard Deviation ($\sigma_{F}$) [Pixels] | 2 | * |
|  | Local Nudge Standard Deviation ($\sigma_{F}$) [Pixels] | 2 | * |
|  | Mean Global Nudge Radius ($\bar{R}$) [Pixels] | 50 | ** |
|  | Mean Local Nudge Radius ($\bar{R}$) [Pixels] | 30 | ** |
|  | Global Nudge Radius Standard Deviation ($\sigma_{R}$) [Pixels] | 2 | * |
|  | Local Nudge Radius Standard Deviation ($\sigma_{R}$) [Pixels] | 2 | * |
| Outputs | No. fused phenotypes per output sketch | 10 |  |
|  | No. Global shape variations | 25 | *** |
|  | No. Local shape variations per contour cluster | 5-10 | *** |

Tentative Default values are suggested. The inputs with high interactive relevance are identified with '*'.
