# Supplementary material for: The perceived value of human-AI collaboration in early shape exploration: An exploratory assessment
Source: PLoS One. 2022 Sep 12;17(9):e0274496. doi: 10.1371/journal.pone.0274496 (PMC9467378; doi:10.1371/journal.pone.0274496)
Supplement: S1 Appendix — (DOCX) [file pone.0274496.s004.docx]

# S1 Appendix. How Shapi works: Sketch representation

This subsection describes the process for generating a population of rough sketches that largely resemble the shape of the input Seed, but where every sketch is unique in its curves and presents slight variations. The user provides a basic digital sketch (Seed) that may be generated with softwares like Adobe Illustrator or Photoshop and by using a digitizer tablet of some kind. This sketch is a raster image and can contain a blue line indicating a symmetry axis, as well as an arbitrary number of red lines indicating boundaries to constrain the exploration interactively (see Fig. 5). Using the ‘*scikit-image*’ library in Python 2.7-3.7, the image can be accessed as a rectangular grid of pixels, with every pixel represented by the RGB color values. By using color saturation thresholds, the pixels that are too light are not considered relevant, the pixels that are blueish are classified as symmetry points, the ones that are reddish as boundary points, and the rest as contour points. All the relevant pixels are stored as XY points, where the horizontal coordinate comes from the column number and the vertical coordinate from the row number.

As illustrated in Fig. S1(a), linear regression is used to characterize the symmetry axis, if any. The mathematics of a 2D Perceptron are used to identify the points that are on either side of the symmetry axis, and to calculate their perpendicular distance to the axis. This is used to flip all the contour points to one side of the axis so that the information from both sides can be used in the symmetric contour. The image representation and internal manipulations are conducted at this one side of the symmetry axis, and then everything is mirrored back to both sides. If a hand-drawn Seed is not perfectly symmetric, or the symmetric axis was not perfectly placed, this functionality of flipping back and forth allows an averaged symmetric contour to be generated (see Fig. 5). Additionally, the symmetry axis may be placed in non-obvious ways, facilitating potentially surprising divergence (see Fig. 6).

To characterize the boundary lines, the points from each boundary are automatically clustered, and linear regressions are fitted to each group. A boundary point is focused, its neighbors are assigned to the same cluster, these neighbors are then focused, and the process continues until there are no more unfocused neighbors, at which point a new boundary cluster is initialized (Fig. S1(a)). The mathematics of a 2D Perceptron are used to detect and correct any transgressor point by displacing it perpendicularly to the nearest boundary. The ‘correct’ side of each boundary is automatically inferred by calculating in which side of the boundary the centroid among all the contour points is.

The contour points are spatially grouped using K-Means Clustering [65]. For every cluster, the most influential high-interest points are identified (Fig. S1(b)) by measuring the proportional variation in XY point-density with a Finite-Difference scheme [66]. Then, a Genetic Algorithm is used to generate a population of individual phenotypic sketches that approximate the contour points. Every individual is composed of a set of cubic Bézier curves (phenotype) and its genotype is encoded as the coordinates of all its Bézier control points. To initialize a Bézier curve in a given cluster (Fig. S1(c)), the fist Bézier control point (P_1_) is selected among the high-interest points with a Roulette-Wheel scheme [67], where the points that concentrate higher XY density variations are more likely to be selected. The last Bézier control point (P_4_) is also selected among the high-interest points of the same cluster with a Roulette Wheel scheme, but here, the ones that are farther from P_1_ are more likely to be selected. The two intermediate control points (P_2_, P_3_) are initially established with linear interpolation between the ends. Lastly, the endpoints are linearly extrapolated outwards (P_1_^’^, P_4_^’^) to reduce the gaps between neighboring curves. This process is repeated for a given number of curves. To improve representation effectiveness, more curves are initialized in clusters with more contour points.

The GA functions (mutation, cross-over, and elitism) [67] are used to partially fit the initialized Bézier curves to the contour points. The GA seeks to minimize a fitness function composed of two Root-Mean-Square-Error (RMSE) deviations (Fig. S1(d)). RMSE1 measures the mean Euclidean distance between every contour point and the nearest phenotypic Bézier point. RMSE2 measures the mean distance between every phenotypic Bézier point and the nearest contour point. The former is for reducing the number of target points that are not being represented by Bézier curves. The latter is for reducing the portions of Bézier curves that are not correlated to the contour points by pushing the Bézier curves toward the contour points. The parents are selected with a Roulette Wheel scheme based on the RMSE fitness, where the most relevant (correlated to the contour points) phenotypes are more likely to produce offspring. A cross-over scheme based on sexual reproduction at the cluster level is used to generate the offspring (Fig. S1(f)). For every cluster of contour points in an offspring, complete Bézier curves can be inherited from either parent. More curves are allocated to the clusters with more contour points. Besides cross-over, mutations are the main engine of evolutionary adaptation. Every time a random number generator surpasses a threshold, any Bézier control point can be displaced in its XY coordinates according to a Gaussian distribution of mutation values (Fig. S1(e)). The top-performing (RMSE fitness) individuals are selected as ‘elites’. To avoid losing the best solutions and focus the exploration in the vicinity of elites, they are cloned, and both groups of elites are passed on to the next generation, one with mutations and the other without them.
