# Supplementary material for: The perceived value of human-AI collaboration in early shape exploration: An exploratory assessment
Source: PLoS One. 2022 Sep 12;17(9):e0274496. doi: 10.1371/journal.pone.0274496 (PMC9467378; doi:10.1371/journal.pone.0274496)
Supplement: S2 Appendix — (DOCX) [file pone.0274496.s005.docx]

# S2 Appendix. How Shapi works: Divergence from a given Seed

The GA produces a series of individual phenotypes with curves that are greatly influenced by the target points but do not fit them perfectly (Sec. S1), and this is used to the advantage of the purpose of this work. Each phenotype suggests slight deviations from the original contour. The GA population has an inherent diversity because the contour Bézier curves go from straight and more constrained during the first GA generation, to curvier and sometimes erratic toward the last. Also, every selection of phenotypes for being fused is stochastic, so every output sketch results from 10 different superimposed phenotypes. Although these aspects make every output sketch unique in its particular curves, they all tend to represent the patterns of the input Seed.

Hence, to introduce the capability for greater divergence, a ‘Nudge’ function is developed. Nudges are stochastic deformations that can affect the whole set of curves that are in the vicinity of the Nudge's center. For every nudge instantiated, a high-interest point is randomly selected as its center, its direction (θ) is sampled from a uniform distribution ($\boldsymbol{\theta}\sim U(0,2\pi)$), and the maximum force (F) and Radius of Influence (R) are sampled from Normal distributions ($\left. \boldsymbol{F}\sim N(\bar{F}, \sigma_{F} \right)$ and $\left. \boldsymbol{R}\sim N(\bar{R}, \sigma_{R} \right)$). A special function is proposed to determine the displacement ($f_{i}$ ) of the $i^{th}$ point in the Nudge’s direction, where $d_{i}$ is the distance from the Nudge’s center:

$$f_{i}= \left\{ \begin{matrix} \frac{F}{2}\left( 1+\cos\left( \frac{d_{i}\pi}{R} \right) \right), if d_{i}<R \\ 0, otherwise \end{matrix} \right\}$$

Fig. S2 illustrates how Nudges work. The points that are closer to the Nudge's center are displaced more. Nudges can be employed at a global or local level (Fig. 4(b)). Global Nudges have their centers originated in any of the high-interest points. They are more frequent and have a larger force and radius, resulting in variations at the level of the overall shape. Local Nudges are originated within a particular cluster and have a more constrained reach.
