# Supplementary material for: The perceived value of human-AI collaboration in early shape exploration: An exploratory assessment
Source: PLoS One. 2022 Sep 12;17(9):e0274496. doi: 10.1371/journal.pone.0274496 (PMC9467378; doi:10.1371/journal.pone.0274496)
Supplement: S3 Appendix — (DOCX) [file pone.0274496.s006.docx]

# S3 Appendix. How Shapi works: Outputs, convergence, and interactive control

Shapi produces two sets of shape variations: Global Exploration and Local Exploration (Fig. 1-bottom). In the Global Exploration, a number of fused sketches are modified through Global Nudges. Every sketch is unique due to the stochastic selection of the individual phenotypes and the Nudges applied to it. Global Exploration can be used to catalyze insight or ideas about the overall shape or particular details anywhere in the contour. In the Local Exploration, a number of variations with Local Nudges are proposed for every contour cluster (Region). Every variation is generated with a new selection of phenotypes that are locally nudged and highlighted with selective emphasis. Local Exploration can be used for more systematic inspections of the different shape regions. These outputs are meant to help the user to converge toward the next Seed by selecting, transforming, integrating, or getting inspired by the variations, and then producing human reinterpretations. Instances like the ones displayed in Fig. 1 are just a few examples of Shapi’s variations, but Shapi can be used to keep generating shape alternatives indefinitely and support a broader and deeper exploration.

An important aspect of the purpose of this work is for the tool to be adaptable to different shapes and user preferences. Aside from drawing the input contour, any symmetry axis, and an arbitrary number of boundary lines, the user can adjust a series of parameters (Table S1). After exploring the capabilities of the tool with different input shapes and purposes (Sec. 5), the parameters identified to be more relevant for interactive adjustment are marked with (*). For instance, if the shape is more complex, the user may increase the number of contour clusters, curves per phenotype, generations, and population size, but adjusting only the first two is found to be enough in many cases. Additionally, this introduces a trade-off between representation accuracy and the program’s run time. The user may adjust the number of variations in the Global Exploration and the variations per cluster in the Local Exploration depending on the limits of practicality and the desired exploration breadth. Moreover, a more ‘aggressive’ divergence can be attained by increasing the Nudge parameters, especially those marked with (**).
